# Evaluation of a hospital-initiated tobacco dependence treatment service: uptake, smoking cessation, readmission and mortality

**DOI:** 10.1186/s12916-024-03353-8

**Published:** 2024-03-25

**Authors:** John Robins, Irem Patel, Ann McNeill, John Moxham, Arran Woodhouse, Gareth Absalom, Buljana Shehu, Geraldine Bruce, Amy Dewar, Alanna Molloy, Stephanie Duckworth Porras, Michael Waring, Andrew Stock, Debbie Robson

**Affiliations:** 1 Nicotine Research Group, Department of Addictions, Institute of Psychiatry, Psychology and Neuroscience, King’s College London, London, UK; 2 Integrated Care, King’s College Hospital NHS Foundation Trust, Denmark Hill, London, UK; 3 King’s College London, London, UK; 4 Integrated Respiratory Team, King’s College Hospital NHS Foundation Trust, Denmark Hill, London, UK; 5 Integrated Local Services, Guy’s and St Thomas’ NHS Foundation Trust, London, UK; 6 Business Intelligence Unit, King’s College Hospital NHS Foundation Trust, Denmark Hill, London, UK; 7 Respiratory Medicine, Guy’s and St Thomas’, NHS Foundation Trust, London, UK; 8 Health Informatics, Guy’s and St Thomas’ NHS Foundation Trust, London, UK

**Keywords:** Smoking cessation, Tobacco dependence treatment, Hospital

## Abstract

**Background:**

The National Health Service in England aims to implement tobacco dependency treatment services in all hospitals by 2024. We aimed to assess the uptake of a new service, adapted from the Ottawa Model of Smoking Cessation, and its impact on 6-month quit rates and readmission or death at 1-year follow-up.

**Methods:**

We conducted a pragmatic service evaluation of a tobacco dependency service implemented among 2067 patients who smoked who were admitted to 2 acute hospitals in London, England, over a 12-month period from July 2020. The intervention consisted of the systematic identification of smoking status, automatic referral to tobacco dependence specialists, provision of pharmacotherapy and behavioural support throughout the hospital stay, and telephone support for 6 months after discharge. The outcomes were (i) patient acceptance of the intervention during admission, (ii) quit success at 6 months after discharge, (iii) death, or (iv) readmission up to 1 year following discharge. Multivariable logistic regression was used to estimate the impact of a range of clinical and demographic variables on these outcomes.

**Results:**

The majority (79.4%) of patients accepted support at the first assessment. Six months after discharge, 35.1% of successfully contacted patients reported having quit smoking. After adjustment, odds of accepting support were 51–61% higher among patients of all non-White ethnicity groups, relative to White patients, but patients of Mixed, Asian, or Other ethnicities had decreased odds of quit success (adjusted odds ratio (AOR) = 0.32, 95%CI = 0.15–0.66). Decreased odds of accepting support were associated with a diagnosis of cardiovascular disease or diabetes; however, diabetes was associated with increased odds of quit success (AOR = 1.88, 95%CI = 1.17–3.04). Intention to make a quit attempt was associated with a threefold increase in odds of quit success, and 60% lower odds of death, compared to patients who did not intend to quit. A mental health diagnosis was associated with an 84% increase in the odds of dying within 12 months.

**Conclusions:**

The overall quit rates were similar to results from Ottawa models implemented elsewhere, although outcomes varied by site. Outcomes also varied according to patient demographics and diagnoses, suggesting personalised and culturally tailored interventions may be needed to optimise quit success.

**Supplementary Information:**

The online version contains supplementary material available at 10.1186/s12916-024-03353-8.

## Background

Tobacco smoking remains the modifiable mortality risk factor that accounts for more years of life lost than any other [1]. In 2019/2020, there were an estimated 506,100 admissions to hospitals in England that were attributable to smoking, costing an estimated £850 m [2]. Admission to hospital provides a critical window of opportunity for intervention, but these opportunities are often missed, and historically, patients who smoke have received little support to quit during a hospital stay [3–5]. However, studies have demonstrated that hospital-initiated tobacco dependence treatment can increase quit attempts and quit success [6–11], and reduce readmission rates and mortality [9, 12]. The most researched is the Ottawa Model for Smoking Cessation (OMSC) which is a targeted opt-out intervention for people who smoke, initiated during their hospital admission.

The OMSC includes the systematic identification on admission of all patients who smoke, followed by brief advice, personalised bedside counselling, and the provision of pharmacotherapy [9]. Patients receive 8 phone calls for 6 months after discharge to assess current smoking status, confidence in remaining smoke-free, and use of cessation support, as well as to facilitate counselling from a smoking cessation nurse specialist in case of relapse to smoking or low confidence about quitting [13]. A real-world effectiveness study of 14 Canadian hospitals found that among the patients who smoked for whom complete data were available, those who received the OMSC intervention were more likely to have quit smoking at 6 months compared to those who did not (35.2% vs 20.4%, *p* < 0.001) [9]. Other opt-out inpatient tobacco cessation models similar to the OMSC have been developed, including the Medical University of South Carolina (MUSC) inpatient tobacco cessation service in the USA [10], and the Conversation, Understand, Replace, Experts and evidence-based treatments (CURE) project in the UK [11]. An evaluation of the MUSC model found that of the 2316 patients reached by phone a month after discharge, 51% of those who had received the full intervention reported not smoking, compared to 27% of those who had not [10]. Similarly, in an evaluation of the CURE project in one hospital in Manchester, England, 61% of eligible patients accepted support, and 525 patients reported that they had stopped smoking at 12 weeks, representing 66% of those completing follow-up at 12 weeks and 22% of the entire cohort of smokers admitted to the hospital [11].

### Sociodemographic and clinical factors that may influence outcomes

A large body of literature has studied the clinical and sociodemographic factors associated with quit success in general population samples, in which increased quit success is associated with older age and higher socioeconomic status, and decreased quit success is associated with higher nicotine dependence and a history of previous unsuccessful quit attempts [14]. There are similar findings from the community stop-smoking services in the UK, with increased quit success at 1 year associated with older age, higher socioeconomic status, lower nicotine dependence, use of varenicline, having a supportive partner, and having more non-smoking friends [15]. There is a need for more research on the clinical and sociodemographic characteristics of sub-populations for whom hospital-initiated smoking cessation interventions may be most effective, and those for whom additional targeted support may be required. A Cochrane review of interventions for smoking cessation in hospitalised patients compared outcomes in diagnostic subgroups and found 22 studies which looked at outcomes for patients with cardiovascular disease (CVD), for whom outcomes were similar to the wider sample of hospitalised patients, but only 5 studies specific to patients with a respiratory diagnosis and 1 from patients with cancer [7]. Hock et al. [16] identified 14 covariates of quit success relevant to analyses of hospital-initiated tobacco dependence interventions, including demographics, individual physical and mental health status, tobacco smoking behaviours, and intervention characteristics. For example, in a subgroup analysis of data from patients who received the OMSC intervention in 1 of 14 Canadian hospitals, Mullen et al. [9] found that the reductions in 2-year mortality rate observed in the wider cohort did not occur among those with a history of mental illness or who lived in rural areas. Similarly, the reductions in 2-year readmission rates observed in the wider cohort were not found among patients with a history of mental illness, or of higher socioeconomic status, or with congestive heart failure [9].

### Current study

Recently, there has been significant new investment within the National Health Service (NHS) in England that focuses on the treatment of tobacco dependency for patients admitted to acute care, psychiatric hospitals, and maternity services, with OMSC and CURE type models of care recommended [17]. Further research is needed to assess the impact of OMSC-based interventions on patient outcomes in the UK and to identify the social, clinical, and treatment factors associated with quit success and consequent reduction in mortality and healthcare burden. A 1-year pilot of a hospital-initiated smoking cessation service based on the OMSC was conducted across acute inpatient wards of two large partner hospitals in Southeast London, England. This study reports the outcomes of this pilot, in terms of (i) patient acceptance of the intervention during hospital admission, (ii) quit success at 6 months after discharge, (iii) rates of readmission or death up to 1 year following discharge, and (iv) the impact of a range of relevant patient characteristics upon these outcomes.

## Methods

### Design and setting

This report is a pragmatic service evaluation using electronic health record data from patients who smoke, admitted to two London acute NHS teaching hospitals between 1 July 2020 and 30 June 2021: (1) a single 52-bed acute emergency care ward (hospital A) and (2) eleven acute medical and surgical wards, containing over 200 beds (hospital B). The two hospital sites were run by 2 different NHS Foundation Trusts. Although separate organisations, they are part of one academic health sciences centre serving similar and neighbouring inner London populations and were jointly funded by one commissioner to pilot the OMSC intervention. Prior to the OMSC pilot, both sites had some form of hospital tobacco dependence treatment in place for several years that offered opt-in support to some, but not all, parts of the hospitals; each organisation configured and funded their services differently, and the hospital staff did not follow up patients after discharge. For this pilot, with additional new funding, both organisations employed two new dedicated tobacco dependence specialists (TDS) to provide the OMSC intervention. Administrative support was also provided by both organisations. Consultants in respiratory medicine and tobacco dependence treatment managers provided oversight and leadership of the implementation of the pilot service in both organisations.

The evaluation was informed by a standardised evaluation framework for hospital-initiated tobacco dependence treatment services [18].

### Ethical approval

As this was a pragmatic service evaluation using anonymised data previously collected as part of a clinical service, ethical approval was not required [19]. Instead, approval for a service evaluation was given by both trusts prior to the start of the study. Data extraction and analysis were in accordance with all relevant guidelines and regulations including the UK General Data Protection Regulation. All patient-identifiable information such as patient name and NHS number was removed prior to analysis, and age was discretised into broad categories.

### Intervention

The pilot intervention was based on the OMSC [9]. In both hospitals, the ward staff aimed to screen the smoking status of all patients on admission, prescribe nicotine replacement therapy (NRT), and automatically refer patients who smoked to a TDS. Patients were supported by the TDSs throughout their hospital stay and by telephone for 6 months after discharge unless they opted out. NRT) was prescribed throughout admission, and a take-home supply for 2 weeks was provided on discharge. Further supplies were provided by the community stop-smoking services; however, this information was not recorded.

**Fig. 1:**
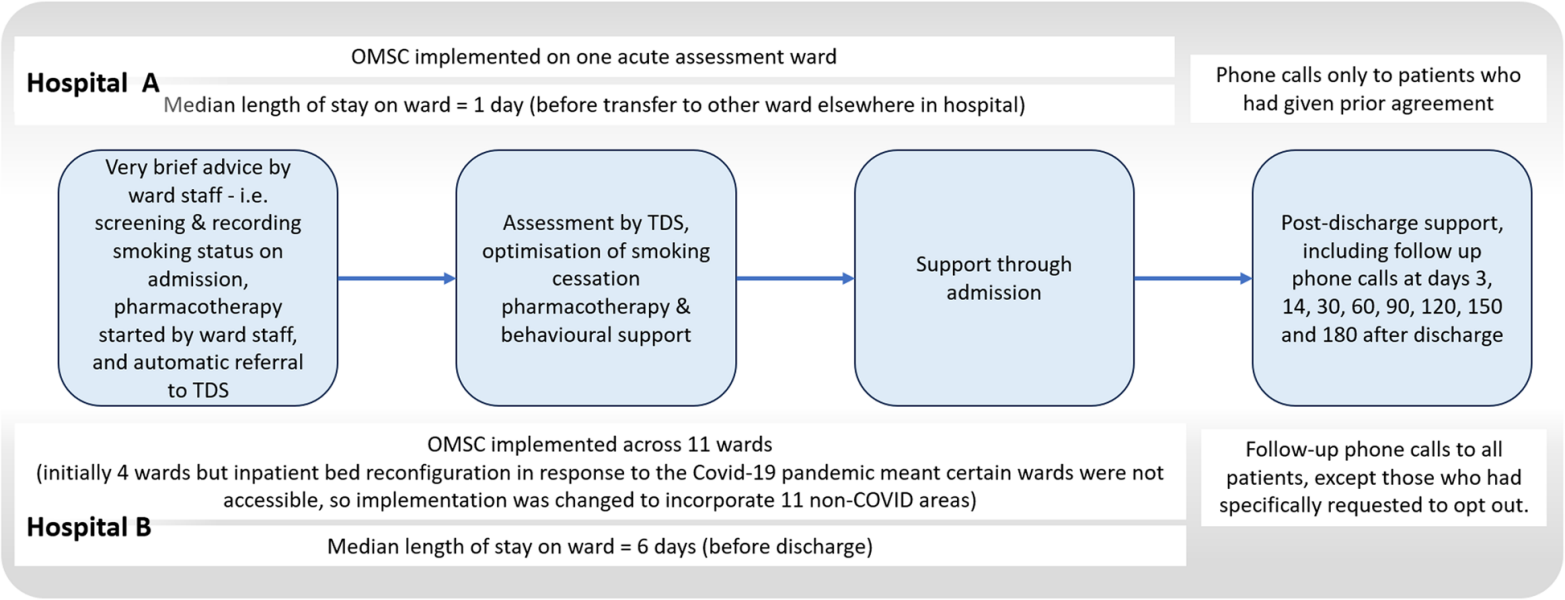
Adapted Ottawa Model of Smoking Cessation. OMSC, Ottawa Model of Smoking Cessation; TDS, tobacco dependence specialists

Follow-up support slightly differed between hospitals. See Fig. 1 for the overview of the intervention and how it was implemented in each site. Hospital A began the implementation of the OMSC in September 2019 and hospital B in January 2020. Some adaptations had to be made from March 2020 onwards, as hospitals reconfigured their services and inpatient wards to respond to the COVID-19 pandemic.

### Participants

**Fig. 2:**
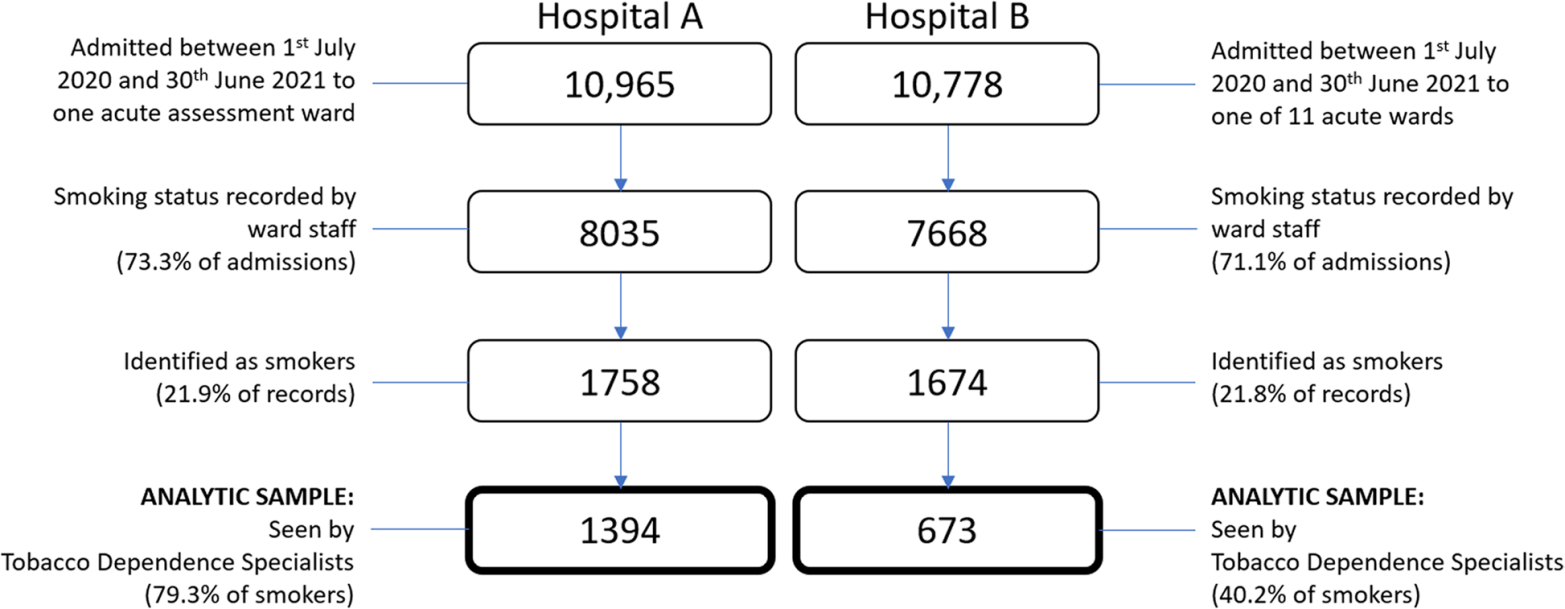
Patient admissions included in evaluation at hospitals A and B

Between 1 July 2020 and 30 June 2021, 72.2% of the 21,743 patients admitted to the participating wards across the 2 acute hospitals had their smoking status recorded by the ward staff, and 3432 (21.9%) were identified as currently smoking. Of these, 2067 (60.2%) were assessed by a TDS and included in the sample for this study. See Fig. 2 for a flow diagram of the patient inclusion frequencies per hospital site. Hospital B had originally planned to provide support on four wards; however, inpatient bed reconfiguration in response to the COVID-19 pandemic meant certain wards were not accessible, so they spread their efforts across 11 non-COVID areas. This resulted in a lower proportion of eligible patients (40.2%) being seen by the TDS compared to hospital A (79.3%) where the intervention remained focused on a single admissions ward.

The 2067 patient admissions presented here comprised 1794 separate individuals; 171 individuals had more than one admission during the study period. The primary analyses are conducted on admission-level data, as many of the clinical covariates and outcomes such as *intention at first TDS assessment* may vary from one admission to the next. However, a sensitivity analysis which restricts the data to the first admissions only is also included.

## Measures

Data were provided by the TDS and the health informatics teams in both organisations.

### Covariates

The following covariates were chosen based on the findings of previous research, e.g. [14–16, 18].

#### Patient demographics

Patient demographics were derived from patients’ admission data, specifically the following: sex; self-reported ethnicity, categorised into White, Black, Asian, Mixed, and Other (see Additional file 1: Table S1 for further detail of ethnicity groupings); and age category on admission, categorised into bands 16–24, 25–39, 40–59, and 60 +, representing youth and three stages of adulthood, respectively. Where the patient was resident in England, the Index of Multiple Deprivation (IMD) score associated with their home postcode was used as a measure of relative socio-economic deprivation. Scores were grouped according to national tertile, with lower scores representing greater levels of deprivation [20].

#### Clinical characteristics of admission

##### Primary diagnosis

Primary diagnoses relating to the presenting problem on admission were grouped according to ICD-10 chapter (A00–B99 infectious and parasitic, C00–D48 neoplasms, E00–E90 endocrine and blood, F00–F99 mental behavioural and neurodevelopmental disorders, G00–G99 nervous system, I00–I99 circulatory, J00–J99 respiratory, K00–K93 digestive, L00–L99 skin and subcutaneous, M00–M99 musculoskeletal, N00–N99 genitourinary, S00–T98 injury poisoning and external causes, and all others) [21]. A binary *smoking-related disease* variable was created which included any condition from chapters relating to circulatory disease, respiratory disease, or neoplasms.

##### Intention at first TDS assessment

Patient intention at the first assessment by a TDS was categorised according to whether they declined the offer of support (*declined intervention*), whether they wished to temporarily abstain with or without the use of smoking cessation aids (*withdrawal management*), or whether they intended to quit (*quit attempt*).

##### Heaviness of Smoking Index (HSI)

Severity of cigarette dependence was measured by the Heaviness of Smoking Index (HSI), a well-validated questionnaire which has been found to be predictive of quit success [22]. The two items of the HSI pertain to the number of cigarettes per day and time to the first cigarette. Respondents are categorised into low (scores 0–1), medium (scores 2–4), or high (scores 5–6) levels of cigarette dependence.

##### Smoking cessation aids

Licensed smoking cessation aids were available and prescribed to patients during admission, comprising NRT in the form of patches, inhalators, mouth spray, lozenges and gum, and varenicline (see Additional file 1: Tables S2 and S3 for an itemised list of prescribed smoking cessation aids). The number of different types prescribed to a patient during admission were grouped as *none*, *single*, or *combination*.

#### Clinical characteristics of patients

##### Past diagnoses

Separate binary variables were used to indicate any previously recorded diagnosis of mental and behavioural disorder (excluding those relating to the use of tobacco), cancer, chronic obstructive pulmonary disease (COPD), cardiovascular disease (CVD), or diabetes.

### Outcomes

#### Outcome 1: Accepted intervention at TDS assessment

A binary variable was created based on the patient’s intention at first TDS assessment, to indicate whether a patient declined the intervention (opted out) or agreed to the intervention (regardless of whether they intended to quit smoking or simply manage their tobacco withdrawal whilst in hospital).

#### Outcome 2: Smoking cessation

Smoking status was recorded at the start of admission and at each phone call during the 6-month post-discharge period. As per previous studies, where a TDS was unable to contact the patient, this was assumed to be indicative of the patient having relapsed or continuing to smoke [8, 11]. Three binary variables were used to indicate a patient’s smoking status (*non-smoker* vs *smoker or unknown*) at 30, 90, and 180 days post-discharge.

#### Outcome 3: All-cause death

A binary variable was created to indicate death by any cause, from 31 days to 1 year after discharge. This timespan was chosen as it permits the inclusion of smoking status at up to 30 days post-discharge as an exposure in regression models, and deaths that occur within 30 days are unlikely to have been influenced by the intervention and/or quit success during and immediately after initial admission.

#### Outcome 4: All-cause readmission

A binary variable was created to indicate readmission for any cause, from 31 days to 1 year after discharge, on the same rationale as outcome 3 (*all-cause death*) above.

### Analysis

All analyses were performed using R version 4.3.0 [23]. Descriptive analyses were conducted using counts and proportions. Quit rates (those defined as non-smokers) at 30, 90, and 180 days were calculated using the numbers seen by TDS as the denominator. Descriptive statistics are reported separately for each hospital site.

Logistic regression models were fit to the data to estimate the association of the covariates described above with each of the outcomes of interest. Adjusted estimates from multivariable models containing all covariates are reported alongside unadjusted estimates. Odds ratios, confidence intervals, and *p*-values are reported for all covariates, representing the direct effect of each covariate when all others are held constant (in the adjusted model). No set threshold for statistical significance was applied to the primary analysis; odds ratios, confidence intervals, and *p*-values were all considered in assessing the magnitude and meaning of the results [24, 25].

Patients who died within 180 days of discharge were removed from the models of smoking status outcomes. Patients who lived outside of London were removed for the analysis of all-cause readmission, on the assumption that such patients would be less likely to be readmitted to the index hospital given other hospitals would be closer to home for such patients. The logistic regression models used combined data from both sites to maximise sample size but included the hospital site as a covariate to control for site-specific effects. The full sample frequencies of each covariate stratified by each outcome are available in Additional file 1: Tables S4–S7, and the frequencies as used in the regression models but separated by each hospital site are available in Additional file 1: Tables S8–S11.

### Missing data

There were missing data in all variables although the extent of missingness varied greatly, from 0.5% missing *sex* (*n* = 10) to 40.4% missing constituent HSI score item *time-to-first-cigarette* (*n* = 835). Missing data were imputed using multiple imputation by chained equations, using the *mice* package in R [26] and following published guidance [27, 28]. All outcomes and covariates from the primary analysis were included in the imputation model in their original form (e.g. component items of the HSI were imputed separately rather than the derived HSI score itself). Where there were auxiliary variables not included in the main analysis but observed in the data provided by both hospitals, these were also included to assist the accuracy of the imputation model. The final imputation model contained 31 variables. Imputed values were generated for all missing data, except for where a *smoking status* outcome was observed as ‘unknown’ as these were retained as a presumptive indication of smoking, as stated above. Fifty imputed data sets were generated. Variable distributions were compared between observed and imputed data (see Additional file 1: Fig. S1). Results from the analyses were combined using Rubin’s rules [28, 29].

### Sensitivity analyses

For smoking status, we also calculated quit rates at 30, 90, and 180 days (i) among those identified as smoking on admission (whether assessed by TDS or not) and (ii) among only those who had complete follow-up data (i.e. where *smoking status* was not recorded as ‘unknown’).

For the regression results, a range of sensitivity analyses were conducted to test the robustness of the primary analysis. For each outcome, effect estimates from the logistic regression using imputed data (the primary analysis) were compared with effect estimates from (i) a complete case analysis which excluded any cases for which there were missing data in any of the variables included in the primary regression model, (ii) a first-admission-only analysis which excluded any repeat admissions for an individual previously admitted during the study period, (iii) a complete case analysis of the first-admission-only data, and (iv) analysis restricted to patients who were seen by the TDS and accepted the intervention, i.e. excluding any patients where the intervention was declined at first assessment.

## Results

### Sample characteristics

In the study period, those seen by the TDS (*n* = 2067, Table 1) across both hospital sites were typically male (*n* = 1334, 64.5%), of white ethnicity (*n* = 1306, 63.2%), aged between 40 and 59 (*n* = 784, 37.9%), and resident in an area in the lower tertile of IMD scores representing higher relative deprivation (*n* = 855, 41.4%). The most common primary diagnosis on admission was in the category *mental and behavioural disorders* (*n* = 190, 13.6%) in hospital A and the category *injury, poisoning, and external causes* in hospital B (*n* = 179, 26.6%). This difference reflects hospital B’s role as a major trauma centre. Prior diagnoses of mental/behavioural disorders were particularly prevalent in the sample, with 66.5% (*n* = 1375) overall having ever had such a diagnosis. The median length of stay on the ward where OMSC was delivered differed between the two sites: 1 day in hospital A and 6 days in hospital B.

**Table 1:** Summary statistics describing sample (*N* = 2067), stratified by hospital site

Variable	Categories	Total		Hospital A		Hospital B		*p*-value
		***N***	**%**	***N***	**%**	***N***	**%**	
**Age on admission**	16–24	126	*6.1*	85	*6.1*	41	*6.1*	0.993
	25–39	427	*20.7*	283	*20.3*	144	*21.4*	
	40–59	784	*37.9*	524	*37.6*	260	*38.6*	
	60 +	679	*32.8*	451	*32.4*	228	*33.9*	
	*Missing*	*51*	*2.5*	*51*	*3.7*	*0*	*0.0*	
**Sex**	Male	1334	*64.5*	912	*65.4*	422	*62.7*	0.170
	Female	723	*35.0*	472	*33.9*	251	*37.3*	
	*Missing*	*10*	*0.5*	*10*	*0.7*	*0*	*0.0*	
**Ethnicity**	Asian	61	*3.0*	48	*3.4*	13	*1.9*	< 0.001
	Black	238	*11.5*	113	*8.1*	125	*18.6*	
	White	1306	*63.2*	917	*65.8*	389	*57.8*	
	Mixed	43	*2.1*	22	*1.6*	21	*3.1*	
	Other	117	*5.7*	54	*3.9*	63	*9.4*	
	Declined or not stated	234	*11.3*	172	*12.3*	62	*9.2*	
	*Missing*	*68*	*3.3*	*68*	*4.9*	*0*	*0.0*	
**IMD tertile**	Lower	855	*41.4*	526	*37.7*	329	*48.9*	< 0.001
	Middle	509	*24.6*	316	*22.7*	193	*28.7*	
	Upper	133	*6.4*	74	*5.3*	59	*8.8*	
	*Missing*	*570*	*27.6*	*478*	*34.3*	*92*	*13.7*	
**HSI category**	Low	348	*16.8*	154	*11.0*	194	*28.8*	< 0.001
	Medium	774	*37.4*	480	*34.4*	294	*43.7*	
	High	108	*5.2*	66	*4.7*	42	*6.2*	
	*Missing*	*837*	*40.5*	*694*	*49.8*	*143*	*21.2*	
**Smoking cessation aids**	None	886	*42.9*	560	*40.2*	326	*48.4*	< 0.001
	Single	470	*22.7*	311	*22.3*	159	*23.6*	
	Combination	700	*33.9*	523	*37.5*	177	*26.3*	
	*Missing*	*11*	*0.5*	*0*	*0*	*11*	*1.6*	
**Smoking-related diagnosis**	Yes	399	*19.3*	246	*17.6*	153	*22.7*	0.866
	No	1257	*60.8*	783	*56.2*	474	*70.4*	
	*Missing*	*411*	*19.9*	*365*	*26.2*	*46*	*6.8*	
**Presenting primary diagnosis**	Injury, poisoning, and external	306	*14.8*	127	*9.1*	179	*26.6*	< 0.001
	Digestive	215	*10.4*	116	*8.3*	99	*14.7*	
	Mental and behavioural	214	*10.4*	190	*13.6*	24	*3.6*	
	Respiratory	205	*9.9*	145	*10.4*	60	*8.9*	
	Circulatory	131	*6.3*	77	*5.5*	54	*8.0*	
	Endocrine and blood	106	*5.1*	67	*4.8*	39	*5.8*	
	Genitourinary	64	*3.1*	33	*2.4*	31	*4.6*	
	Neoplasms	63	*3.0*	24	*1.7*	39	*5.8*	
	Infectious and parasitic	49	*2.4*	23	*1.6*	26	*3.9*	
	Musculoskeletal	47	*2.3*	27	*1.9*	20	*3.0*	
	Nervous system	46	*2.2*	31	*2.2*	15	*2.2*	
	Skin and subcutaneous	37	*1.8*	25	*1.8*	12	*1.8*	
	Others	173	*8.4*	144	*10.3*	29	*4.3*	
	*Missing*	*411*	*19.9*	*365*	*26.2*	*46*	*6.8*	
**Past diagnoses**	Cancer	217	*10.5*	179	*12.8*	38	*5.6*	< 0.001
	COPD	557	*26.9*	402	*28.8*	155	*23.0*	0.006
	CVD	310	*15.0*	234	*16.8*	76	*11.3*	0.001
	Diabetes	438	*21.2*	367	*26.3*	71	*10.5*	< 0.001
	Mental and behavioural	1375	*66.5*	1039	*74.5*	336	*49.9*	< 0.001
**Intention at first TDS assessment**	Patient declined	407	*19.7*	340	*24.4*	67	*10.0*	< 0.001
	Withdrawal management	1100	*53.2*	805	*57.7*	295	*43.8*	
	Quit attempt	542	*26.2*	242	*17.4*	300	*44.6*	
	*Missing*	*18*	*0.9*	*7*	*0.5*	*11*	*1.6*	

*IMD* Index of Multiple Deprivation (only available for patients resident in England), *HSI* Heaviness of Smoking Index, *TDS* tobacco dependence specialists

**Fig. 3:**
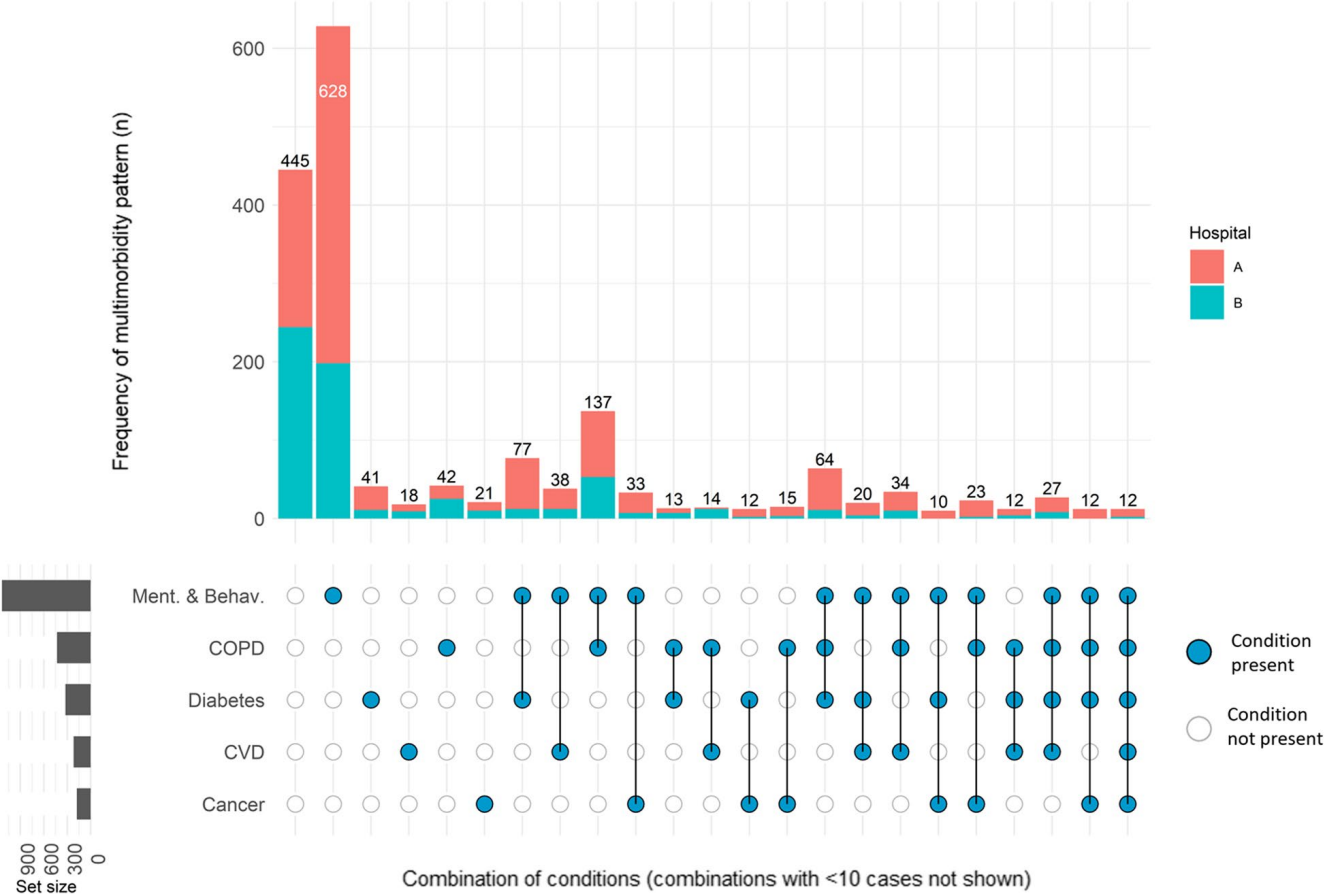
Multimorbidity UpSet plot. The plot shows the frequencies of all combinations of co-occurring mental health and smoking-related chronic physical conditions in the cohort, according to recorded past diagnoses. Restricted to first admission only (*N* = 1794). The dots represent the presence of a condition, ranging from all five conditions absent on the far left to all five conditions present on the far right, with all commonly co-occurring combinations of conditions (i.e. > 9 cases in cohort) in between. Mental illness includes all conditions from chapter F of the ICD-10, excluding diagnoses pertaining to nicotine dependence (F17). Frequencies fewer than 10 suppressed to prevent deanonymisation

Multimorbidity of smoking-related long-term conditions and mental ill-health was common. Seventy-five per cent (*n* = 1349) of the cohort had a past diagnosis of at least one of CVD, COPD, diabetes, cancer, or a mental health condition (*n* = 934 [82.3%] in hospital A and *n* = 415 [63.0%] in hospital B), and 33.3% (*n* = 599) had at least two of these conditions (*n* = 437 [38.5%] in hospital A and *n* = 162 [24.6%] in hospital B). Figure 3 shows the overall frequencies of each combination of these conditions, with each bar split by hospital. At both hospital sites, the most frequently co-occurring pair of conditions was a mental/behavioural disorder and COPD (*n* = 84 [7.4%] at hospital A and *n* = 53 [8.0%] at hospital B), and the most frequent co-occurring trio of conditions added diabetes to this (*n* = 53 [4.7%] at hospital A and *n* = 11 [1.7%] at hospital B).

#### Outcome 1: Accepted intervention at TDS assessment

Overall, the OMSC intervention was accepted by 1642 patients (79.4%) (Table 1). The offer of support was accepted by a greater proportion of patients in hospital B (*n* = 595 [88.4%]) than in hospital A (*n* = 1047 [75.1%]), and a quit attempt was made by a greater proportion of patients in hospital B (*n* = 300 [44.6%]) than in hospital A (*n* = 242 [17.4%]).

After adjustment, increased odds of accepting the OMSC intervention at first TDS assessment were associated with Black ethnicity (adjusted odds ratio (AOR) 1.61; 95%CI 1.06, 2.44; *p* = 0.025) and Mixed/Asian/Other ethnicities (AOR 1.51; 95%CI 1.00, 2.28; *p* = 0.048) compared to White ethnicity, as well as being admitted for a smoking-related condition (i.e. cancer, respiratory, or circulatory disease) (AOR 1.41; 95%CI 1.02, 1.95; *p* = 0.035). After adjustment, reduced odds of accepting the OMSC intervention were associated with being in the youngest age bracket (16–24 years) compared to patients aged 60 or over (AOR 0.50; 95%CI 0.30, 0.83; *p* = 0.007), as well as having a history of CVD (AOR 0.64; 95%CI 0.46, 0.89; *p* = 0.007) or diabetes (AOR 0.73; 95%CI 0.55, 0.96; *p* = 0.026). Table 2 presents odds ratios for all covariates in the model.

**Table 2:** Logistic regression model for outcome 1: accepted intervention at first TDS assessment (*n* = 2067)

	**Category**	**Unadjusted OR (95%CI)**	***p*-value**	**Adjusted OR (95%CI)**	***p*-value**
**Accepted intervention at first TDS assessment**					
**Hospital**	**A**	(Ref.)		(Ref.)	
	**B**	**2.88 (2.19–3.85)**	** < 0.001**	**2.63 (1.95–3.54)**	** < 0.001**
**Age on admission**	**60 + **	(Ref.)		(Ref.)	
	**40–59**	0.90 (0.69–1.18)	0.448	0.83 (0.61–1.12)	0.222
	**25–39**	0.83 (0.61–1.12)	0.229	0.76 (0.52–1.10)	0.149
	**16–24**	**0.53 (0.35–0.82)**	**0.004**	**0.50 (0.30–0.83)**	**0.007**
**Sex**	**Male**	(Ref.)		(Ref.)	
	**Female**	1.07 (0.85–1.34)	0.577	1.00 (0.79–1.28)	0.970
**Ethnicity**	**White**	(Ref.)		(Ref.)	
	**Black**	**1.73 (1.17–2.55)**	**0.006**	**1.61 (1.06–2.44)**	**0.025**
	**Mixed/Asian/Other**^a^	1.38 (0.94–2.03)	0.104	**1.51 (1.00–2.28)**	**0.048**
**HSI category**	**Low**	(Ref.)		(Ref.)	
	**Medium**	1.10 (0.82–1.47)	0.541	1.17 (0.85–1.62)	0.331
	**High**	1.21 (0.71–2.07)	0.473	1.32 (0.75–2.30)	0.331
**Primary diagnosis**	**Smoking-related**	**1.56 (1.15–2.10)**	**0.004**	**1.41 (1.02–1.95)**	**0.035**
**Past diagnoses**	**Cancer**	0.88 (0.63–1.26)	0.483	0.91 (0.63–1.33)	0.630
	**COPD**	1.21 (0.95–1.56)	0.134	1.30 (0.96–1.77)	0.091
	**CVD**	**0.67 (0.51–0.90)**	**0.006**	**0.64 (0.46–0.89)**	**0.007**
	**Diabetes**	**0.67 (0.52–0.86)**	**0.001**	**0.73 (0.55–0.96)**	**0.026**
	**Mental and behav**	0.86 (0.68–1.09)	0.220	1.08 (0.84–1.40)	0.540
**IMD tertile**	**Lower**	(Ref.)		(Ref.)	
	**Middle**	0.90 (0.68–1.20)	0.471	0.95 (0.71–1.28)	0.755
	**Upper**	0.77 (0.50–1.18)	0.228	0.81 (0.51–1.28)	0.365

Unadjusted and fully adjusted estimates from logistic regression model for outcome: accepted intervention at first TDS assessment, using imputed analysis (*n* = 2067)

Adjusted model adjusted for all variables in the table

*IMD* Index of Multiple Deprivation, *HSI* Heaviness of Smoking Index, *CVD* cardiovascular disease

^a^Categories collapsed due to sparse data

#### Outcome 2: Smoking status

Overall quit rates and by hospital site are reported in Table 3, using the number of patients assessed by a TDS as the denominator (excluding patients who had died). Many patients were unable to be contacted post-discharge; at 30 days, 29.6% (*n* = 601/2030) of patients were successfully contacted, falling to 22% (*n* = 433/1957) at 180 days. On the assumption that patients who were not contactable during follow-up had relapsed or continued to smoke, 9.6% (*n* = 195) of the overall cohort self-reported they had quit smoking at 30 days post-discharge, 8.4% (*n* = 170) at 90 days post-discharge, and 7.8% (*n* = 152) self-reported they had quit at 180 days post-discharge.

**Table 3:** Quit rates at 30, 90, and 180 days post-discharge

*Days post-discharge*	*Denominator: smokers assessed by TDS (excl. pts who died)*		
	*Total non-smoker*	*Hospital A*	*Hospital B*
*30 days*	195/2030 (9.6%)	110/1371 (8.0%)	85/659 (12.9%)
*90 days*	170/2026 (8.4%)	88/1371 (6.4%)	82/655 (12.5%)
*180 days*	152/1957 (7.8%)	48/1306 (3.7%)	104/651 (16.0%)

The table shows the number of patients reporting being a non-smoker/number of patients assessed by TDS (and expressed as a percentage). Patients who died are excluded from the denominator and the corresponding percentage

*TDS* tobacco dependence specialist

**Fig. 4:**
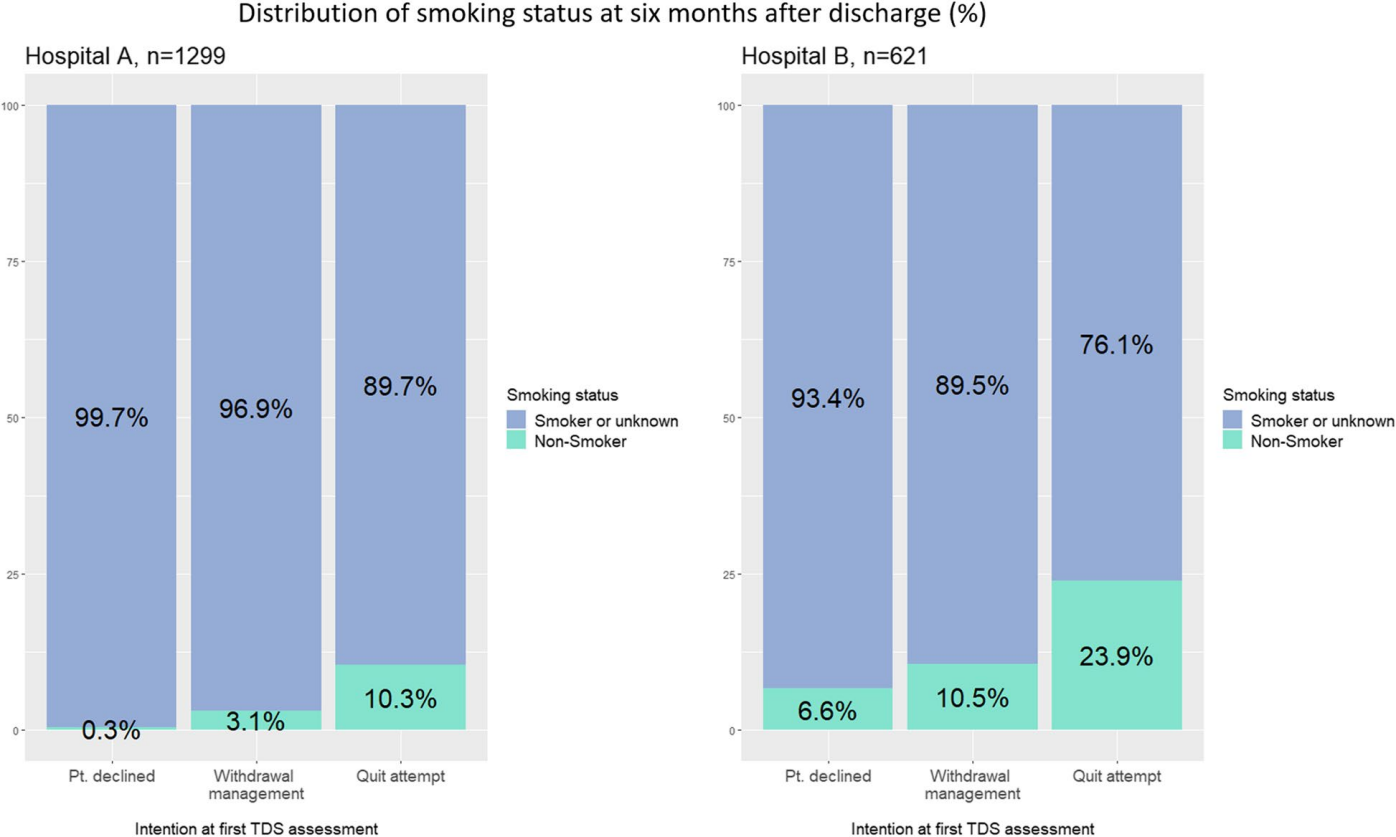
Distribution of smoking status at 180 days after discharge, by quit intention at first TDS assessment. Patients with missing intention information and patient deaths within 180 days excluded

In both hospitals, the proportion of patients reporting being a non-smoker at 180 days was greatest among those whose intention at first TDS assessment was to make a quit attempt; overall, 17.8% (*n* = 92) of patients who intended to make a quit attempt reported being a non-smoker at 180 days, compared to 5.1% (*n* = 52) of those whose intention was withdrawal management whilst in hospital, and 1.3% (*n* < 10) of patients who declined the intervention at first TDS assessment. Figure 4 shows a stacked bar graph comparing quit rates at 180 days after discharge, according to the patient’s quit intention and hospital site.

After adjustment in the regression analysis, the greatest increase in odds of being a non-smoker at 180 days post-discharge was associated with intention to make a quit attempt, compared to only wanting withdrawal management or declining the intervention (AOR 3.68; 95%CI 2.48, 5.48; *p* < 0.001). Significant increases were also associated with a past diagnosis of diabetes (AOR 1.88; 95%CI 1.17, 3.04; *p* = 0.009) and being admitted for a smoking-related condition (AOR 1.76; 95%CI 1.14, 2.71; *p* = 0.010). After adjustment, decreased odds of reporting being a non-smoker at 180 days post-discharge were associated with medium (HSI scores 2–4) or high (HSI scores 5–6) cigarette dependence, compared to low cigarette dependence (AOR 0.45; 95%CI 0.29, 0.69; *p* < 0.001, and AOR 0.42; 95%CI 0.18, 0.99; *p* = 0.048, respectively), Mixed/Asian/Other ethnicities compared to White ethnicity (AOR 0.32; 95%CI 0.15,v0.66; *p* = 0.002), and being aged 16–24 compared to patients aged 60 or over (AOR 0.36; 95%CI 0.13, 1.00; *p* = 0.05). Table 4 presents the odds ratios for all covariates in the model. See Additional file 1: Tables S12 and S13 for odds ratios relating to smoking status outcomes at 30 and 90 days post-discharge.

**Table 4:** Logistic regression model for outcome 2: smoking status (non-smoker) at 180 days post-discharge (*n* = 1957)

	**Category**	**Unadjusted OR (95%CI)**	***p*-value**	**Adjusted OR (95%CI)**	***p*-value**
**Smoking status 180 days**					
**Hospital**	**A**	(Ref.)		(Ref.)	
	**B**	**5.17 (3.64–7.45)**	** < 0.001**	**3.48 (2.30–5.28)**	** < 0.001**
**Age on admission**	**60 + **	(Ref.)		(Ref.)	
	**40–59**	**0.57 (0.39–0.84)**	**0.004**	0.74 (0.47–1.16)	0.185
	**25–39**	**0.60 (0.38–0.94)**	**0.026**	0.82 (0.47–1.44)	0.483
	**16–24**	**0.33 (0.13–0.85)**	**0.021**	**0.36 (0.13–1.00)**	**0.050**
**Sex**	**Male**	(Ref.)		(Ref.)	
	**Female**	1.06 (0.75–1.50)	0.734	0.92 (0.63–1.35)	0.674
**Ethnicity**	**White**	(Ref.)		(Ref.)	
	**Black**	**1.69 (1.10–2.60)**	**0.017**	1.00 (0.60–1.67)	0.997
	**Mixed/Asian/Other**^a^	**0.47 (0.24–0.94)**	**0.034**	**0.32 (0.15–0.66)**	**0.002**
**Intention at first TDS assessment**	**Declined/withdrawal management**^a^	(Ref.)		(Ref.)	
	**Quit attempt**	**5.06 (3.58–7.16)**	** < 0.001**	**3.68 (2.48–5.48)**	** < 0.001**
**Heaviness of Smoking Index**	**Low**	(Ref.)		(Ref.)	
	**Medium**	**0.42 (0.29–0.60)**	** < 0.001**	**0.45 (0.29–0.69)**	** < 0.001**
	**High**	**0.35 (0.16–0.77)**	**0.009**	**0.42 (0.18–0.99)**	**0.048**
**Smoking cessation aids**	**None**	(Ref.)		(Ref.)	
	**Single**	0.78 (0.51–1.20)	0.265	0.74 (0.46–1.18)	0.207
	**Combination**	0.69 (0.47–1.02)	0.060	0.86 (0.53–1.39)	0.529
**Primary diagnosis**	**Smoking-related**	**2.34 (1.63–3.36)**	** < 0.001**	**1.76 (1.14–2.71)**	**0.010**
**Past diagnoses**	**Cancer**	0.73 (0.35–1.35)	0.352	0.60 (0.29–1.25)	0.172
	**COPD**	1.16 (0.80–1.67)	0.421	0.85 (0.53–1.37)	0.506
	**CVD**	1.50 (0.96–2.26)	0.064	1.40 (0.83–2.35)	0.207
	**Diabetes**	1.28 (0.85–1.87)	0.221	**1.88 (1.17–3.04)**	**0.009**
	**Mental and behav**	**0.45 (0.32–0.62)**	** < 0.001**	0.81 (0.55–1.19)	0.277
**IMD tertile**	**Lower**	(Ref.)		(Ref.)	
	**Middle**	1.04 (0.70–1.54)	0.853	1.24 (0.80–1.92)	0.343
	**Upper**	1.05 (0.55–2.03)	0.876	0.98 (0.46–2.07)	0.956

Unadjusted and fully adjusted estimates from logistic regression model for outcome 2: non-smoker at 180 days post-discharge, using imputed analysis, excluding patients who died within 180 days of discharge (*n* = 1957). Adjusted model adjusted for all variables in the table

*IMD* Index of Multiple Deprivation, *HSI* Heaviness of Smoking Index, *CVD* cardiovascular disease

^a^Categories collapsed due to sparse data

#### Outcome 3: All-cause death

Between 31 days and 1 year post-discharge, 104 patients (5.8%) died (73 from hospital A [6.4%] and 31 from hospital B [4.7%]). After adjustment, increased odds of death by any cause between 31 days and 1 year post-discharge were associated with a history of cancer (AOR 3.91; 95%CI 2.41, 6.35; *p* < 0.001), diabetes (AOR 1.74; 95%CI 1.08, 2.81; *p* = 0.023), or mental/behavioural disorder (AOR 1.84; 95%CI 1.11, 3.05; *p* = 0.018), and where present admission was due to a smoking-related condition (AOR 2.87; 95%CI 1.82, 4.53; *p* < 0.001). After adjustment, decreased odds of death by any cause between 31 days and 1 year post-discharge were associated with being younger than 60, either within the age bracket 16–39 (AOR 0.08; 95%CI 0.02, 0.28; *p* < 0.001) or 40–59 (AOR 0.54; 95%CI 0.33, 0.89; *p* = 0.015), female sex (AOR 0.52; 95%CI 0.32, 0.84; *p* = 0.008), and intention at first TDS assessment to make a quit attempt, compared to wanting withdrawal management or declining the intervention (AOR 0.4; 95%CI 0.22, 0.73; *p* = 0.003). Being a non-smoker at 30 days was not associated with greater or lower odds of death by any cause between 31 days and 1 year post-discharge. See Table 5 for the odds ratios for all covariates in the model.

**Table 5:** Logistic regression model for outcome 3: all-cause death between 31 days and 1 year after discharge (*n* = 1770)

	**Category**	**Unadjusted OR (95%CI)**	***p*-value**	**Adjusted OR (95%CI)**	***p*-value**
**Death 31 days to 1 year**					
**Hospital**	**A**	(Ref.)		(Ref.)	
	**B**	0.65 (0.41–0.98)	0.047	1.08 (0.65–1.80)	0.773
**Age on admission**	**60 + **	(Ref.)		(Ref.)	
	**40–59**	**0.36 (0.24–0.55)**	** < 0.001**	**0.54 (0.33–0.89)**	**0.015**
	**16–39**^a^	**0.04 (0.01–0.13)**	** < 0.001**	**0.08 (0.02–0.28)**	** < 0.001**
**Sex**	**Male**	(Ref.)		(Ref.)	
	**Female**	**0.57 (0.36–0.89)**	**0.014**	**0.52 (0.32–0.84)**	**0.008**
**Ethnicity**	**White**	(Ref.)		(Ref.)	
	**Black**	0.49 (0.23–1.02)	0.058	0.55 (0.24–1.25)	0.154
	**Mixed/Asian/Other**^a^	0.59 (0.30–1.17)	0.129	0.94 (0.44–2.00)	0.874
**Intention at first TDS assessment**	**Declined/withdrawal management**^a^	(Ref.)		(Ref.)	
	**Quit attempt**	**0.45 (0.27–0.77)**	**0.003**	**0.40 (0.22–0.73)**	**0.003**
**Heaviness of Smoking Index**	**Low**	(Ref.)		(Ref.)	
	**Medium/high**^a^	1.25 (0.74–2.12)	0.404	0.84 (0.45–1.55)	0.572
**Smoking cessation aids**	**None**	(Ref.)		(Ref.)	
	**Single**	0.91 (0.54–1.51)	0.703	0.88 (0.50–1.55)	0.656
	**Combination**	1.02 (0.66–1.58)	0.938	1.04 (0.62–1.76)	0.873
**Primary diagnosis**	**Smoking-related**	**3.63 (2.43–5.41)**	** < 0.001**	**2.87 (1.82–4.53)**	** < 0.001**
**Past diagnoses**	**Cancer**	**6.83 (4.42–10.44)**	** < 0.001**	**3.91 (2.41–6.35)**	** < 0.001**
	**COPD**	**2.72 (1.82–4.03)**	** < 0.001**	0.85 (0.53–1.37)	0.505
	**CVD**	**3.09 (1.95–4.78)**	** < 0.001**	1.17 (0.70–1.96)	0.556
	**Diabetes**	**3.17 (2.10–4.75)**	** < 0.001**	**1.74 (1.08–2.81)**	**0.023**
	**Mental and behav**	**1.72 (1.12–2.72)**	**0.016**	**1.84 (1.11–3.05)**	**0.018**
**IMD tertile**	**Lower**	(Ref.)		(Ref.)	
	**Middle**	0.73 (0.46–1.16)	0.178	0.75 (0.45–1.25)	0.268
	**Upper**	**0.35 (0.13–0.97)**	**0.044**	0.40 (0.13–1.20)	0.103
**Smoking status 30 days**	**Smoker or unknown**^b^	(Ref.)		(Ref.)	
	**Non-smoker**	0.94 (0.48–1.84)	0.859	1.41 (0.65–3.05)	0.378

Unadjusted and fully adjusted estimates from logistic regression model for outcome 3: all-cause death between 31 days and 1 year after discharge, using imputed analysis restricted to first admission only and excluding patients who died within 30 days of discharge (*n* = 1770). Adjusted model adjusted for all variables in the table

*IMD* Index of Multiple Deprivation, *HSI* Heaviness of Smoking Index, *CVD* cardiovascular disease

^a^Categories collapsed due to sparse data

^b^Patients with ‘unknown’ smoking status were presumed to be smokers, as per previous studies [8, 11]

#### Outcome 4: All-cause readmission

Between 31 days and 1 year post-discharge, 462 patients (22.4%) were readmitted (352 from hospital A (31.0%) and 110 from hospital B [16.7%]). After adjustment, increased odds of all-cause readmission between 31 days and 1 year post-discharge were associated with being aged 40–59, compared to those aged 60 or over (AOR 1.38; 95%CI 1.02, 1.88; *p* = 0.04), being prescribed a single smoking cessation aid compared to no prescription (AOR 1.46; 95%CI 1.06, 2.01; *p* = 0.022), and a history of mental/behavioural disorder (AOR 2.69; 95%CI 1.98, 3.63; *p* < 0.001), COPD (AOR 2.00; 95%CI 1.48, 2.71; *p* < 0.001), CVD (AOR 2.00; 95%CI 1.40, 2.84; *p* < 0.001), cancer (AOR 1.65; 95%CI 1.09, 2.51; *p* = 0.018), or diabetes (AOR 1.41; 95%CI 1.03, 1.92; *p* = 0.032). Being a non-smoker at 30 days was not associated with greater or lower odds of readmission between 31 days and 1 year post-discharge. See Table 6 for the odds ratios for all covariates in the model.

**Table 6:** Logistic regression model for outcome 4: all-cause readmission between 31 days and 1 year after discharge (*n* = 1534)

	**Category**	**Unadjusted OR (95%CI)**	***p*-value**	**Adjusted OR (95%CI)**	***p*-value**
**Readmission 31 days to 1 year**					
**Hospital**	**A**	(Ref.)		(Ref.)	
	**B**	**0.47 (0.36–0.61)**	** < 0.001**	**0.68 (0.51–0.92)**	**0.012**
**Age on admission**	**60 + **	(Ref.)		(Ref.)	
	**40–59**	0.93 (0.72–1.21)	0.596	**1.38 (1.02–1.88)**	**0.040**
	**25–39**	**0.45 (0.32–0.63)**	** < 0.001**	0.87 (0.57–1.32)	0.515
	**16–24**	**0.44 (0.25–0.78)**	**0.005**	0.91 (0.48–1.75)	0.785
**Sex**	**Male**	(Ref.)		(Ref.)	
	**Female**	1.10 (0.87–1.38)	0.448	1.09 (0.84–1.41)	0.508
**Ethnicity**	**White**	(Ref.)		(Ref.)	
	**Black**	0.81 (0.57–1.14)	0.225	1.11 (0.76–1.63)	0.582
	**Mixed/Asian/Other**^a^	0.78 (0.55–1.12)	0.175	1.09 (0.73–1.64)	0.670
**Intention at first TDS assessment**	**Declined/withdrawal management**^**1**^	(Ref.)		(Ref.)	
	**Quit attempt**	0.81 (0.63–1.06)	0.119	0.93 (0.68–1.27)	0.644
**Heaviness of Smoking Index**	**Low**	(Ref.)		(Ref.)	
	**Medium**	1.16 (0.88–1.53)	0.293	0.80 (0.57–1.12)	0.194
	**High**	1.09 (0.65–1.81)	0.743	0.70 (0.39–1.26)	0.229
**Smoking cessation aids**	**None**	(Ref.)		(Ref.)	
	**Single**	**1.46 (1.09–1.97)**	**0.012**	**1.46 (1.06–2.01)**	**0.022**
	**Combination**	**1.60 (1.23–2.08)**	** < 0.001**	1.33 (0.97–1.82)	0.079
**Primary diagnosis**	**Smoking-related**	**1.43 (1.08–1.91)**	**0.013**	1.24 (0.90–1.71)	0.197
**Past diagnoses**	**Cancer**	**2.84 (1.78–4.55)**	** < 0.001**	**1.65 (1.09–2.51)**	**0.018**
	**COPD**	**3.23 (2.45–4.26)**	** < 0.001**	**2.00 (1.48–2.71)**	** < 0.001**
	**CVD**	**2.83 (1.99–4.01)**	** < 0.001**	**2.00 (1.40–2.84)**	** < 0.001**
	**Diabetes**	**2.35 (1.72–3.20)**	** < 0.001**	**1.41 (1.03–1.92)**	**0.032**
	**Mental and behav**	**3.08 (2.28–4.23)**	** < 0.001**	**2.69 (1.98–3.63)**	** < 0.001**
**IMD tertile**	**Lower**	(Ref.)		(Ref.)	
	**Middle**	0.85 (0.64–1.12)	0.248	0.90 (0.67–1.22)	0.504
	**Upper**	0.71 (0.43–1.17)	0.183	0.84 (0.49–1.44)	0.529
**Smoking status 30 days**	**Smoker or unknown**^b^	(Ref.)		(Ref.)	
	**Non-smoker**	0.76 (0.50–1.17)	0.216	0.83 (0.51–1.35)	0.461

Unadjusted and fully adjusted estimates from logistic regression model for outcome 4: all-cause readmission between 31 days and 1 year after discharge, using imputed analysis and excluding patients who died within a year of discharge, or who were readmitted within 30 days, as well as patients resident outside of London (*n* = 1534). Adjusted model adjusted for all variables in the table

*IMD* Index of Multiple Deprivation, *HSI* Heaviness of Smoking Index, *CVD* cardiovascular disease

^a^Categories collapsed due to sparse data

^b^Patients with ‘unknown’ smoking status were presumed to be smokers, as per previous studies [8, 11]

## Sensitivity analyses

### Quit rates by different denominators

Table 7 compares the quit rates at 30, 90, and 180 days from the primary analysis with quit rates calculated as a proportion of all smokers and as a proportion of the cohort for whom follow-up data was complete.

**Table 7:** Sensitivity analysis: quit rates

*Days post-discharge*	*Site*	*Sensitivity analysis 1*	*Primary analysis*	*Sensitivity analysis 2*
		*Denominator* = *a* *ll identified smokers admitted (N* = *3432)*	*Denominator* = *smokers assessed by TDS* *(excl. pts who died)*	*Denominator* = *o* *nly those successfully contacted at follow-up*
*30 days*	Total	195/3432 (5.7%)	195/2030 (9.6%)	195/601 (32.4%)
	Hospital A	110/1758 (6.3%)	110/1371 (8.0%)	110/312 (35.3%)
	Hospital B	85/1674 (5.1%)	85/659 (12.9%)	85/289 (29.4%)
*90 days*	Total	170/3432 (5.0%)	170/2026 (8.4%)	170/466 (36.5%)
	Hospital A	88/1758 (5.0%)	88/1371 (6.4%)	88/217 (40.6%)
	Hospital B	82/1674 (4.9%)	82/655 (12.5%)	82/249 (32.9%)
*180 days*	Total	152/3432 (4.4%)	152/1957 (7.8%)	152/433 (35.1%)
	Hospital A	48/1758 (2.7%)	48/1306 (3.7%)	48/134 (35.8%)
	Hospital B	104/1674 (6.2%)	104/651 (16.0%)	104/299 (34.8%)

Quit rates among (i) all smokers, (ii) those assessed by TDS (as per primary analysis, excluding patients who died), and (iii) patients successfully followed up

### Regression analyses

Each regression model was re-specified to include (i) complete cases only, (ii) first admissions only, (iii) complete case first admissions only, and (iv) only patients who accepted the intervention at the first TDS assessment. Plots comparing the odds ratios and confidence intervals across the primary and sensitivity analyses for all outcomes are provided in Additional file 1: Figs. S2–S7. The direction of effect for each significant covariate in the primary analyses was consistent across all related sensitivity analyses, and all confidence intervals overlapped. The sample sizes of the complete case analyses were between 39 and 65% smaller than those of the corresponding primary analyses, resulting in wider confidence intervals across all estimates, some of which now crossed the null value where they had not done so in the primary analyses. The significant effects identified in the primary analyses which retained odds ratios and confidence intervals entirely outside of the null across all sensitivity analyses were as follows:

- Intention to make a quit attempt, compared to wanting withdrawal management or declining the intervention, which was associated with increased odds of being a non-smoker at 180 days, and lower odds of death between 31 days and 1 year post-discharge
- Medium nicotine dependence (HSI scores 2–4), compared to low nicotine dependence, which was associated with decreased odds of being a non-smoker at 180 days
- Ethnicity categorised as Mixed, Asian, or Other, which was associated with decreased odds of being a non-smoker at 180 days
- Being aged 16–39 (compared to those aged over 60), or being female, which were both associated with reduced odds of death between 31 days and 1 year post-discharge
- A diagnosis of cancer, diabetes, or mental/behavioural disorder, which were all associated with increased odds of death between 31 days and 1 year post-discharge
- A diagnosis of cancer, COPD, CVD, or mental/behavioural disorder, which were all associated with increased odds of readmission between 31 days and 1 year post-discharge

## Discussion

Over a 12-month period, among the cohort of 2067 patients who smoked and were assessed by a TDS during admission to one of two major acute London teaching hospitals, the majority (79%) accepted tobacco dependence treatment based on the Ottawa Model of Smoking Cessation (OMSC). At 6 months post-discharge, approximately 8% self-reported they had quit smoking (just over a third of the subsample who were successfully contacted during follow-up).

There was a significant difference in the outcomes between the two hospitals, with hospital B having a greater proportion of patients intending to make a quit attempt (44.6% vs 17.4%), and a correspondingly greater proportion reporting being a non-smoker at all time points, with the greatest difference manifesting at 6 months post-discharge (16.0% vs 3.7%). These differences in the outcomes likely reflect the differences in the implementation of the OMSC between the two sites, in particular, the longer median length of stay on the ward where the OMSC was provided (6 days in hospital B vs 1 day in hospital A). It is plausible that a longer hospital stay provides the opportunity for the staff and patients to build a therapeutic relationship and for the patient to engage with the offer of support, including the opportunity to change their mind if they initially declined the intervention. Other implementation differences, such as the protocol for follow-up phone calls (wherein hospital A only made calls to patients who had consented to follow-up, but hospital B contacted all patients unless they had specifically requested to opt-out), may have also influenced quit success. However, TDS at hospital A were able to assess a greater proportion of the admitted patients who smoked than at hospital B (79.3% vs 40.2%), likely due to efforts being focused on the single ward. Hospital B also had proportionally fewer readmissions of patients, but this may reflect hospital B’s role as a major trauma centre.

It is important to note that in this study, the OMSC intervention was piloted during the first year of the COVID-19 pandemic, a time of unprecedented disruption to hospital services, and comparisons with the outcomes of previous evaluations of hospital-initiated tobacco dependence treatment need to take this into account. The rate of acceptance of support in our study was both higher [11] and lower [10] than in previous studies. The 6-month quit rate (35.1%) among patients who were successfully contacted during follow-up was almost identical to the rate (35.2%) reported by Mullen et al. [9] for the sample of patients who received the OMSC intervention and had available data. Among all patients assessed by a TDS, the overall quit rate in this study (7.8%) was lower than that reported by Mullen et al. (12%), though higher in one of our hospitals, hospital B (16%). Quit rates in our sample at 90 days and 30 days were lower than those reported by Evison et al. [11] and similar to those reported by Nahhas et al. [10].

Although comprising just under 11% of the cohort, patients of Mixed, Asian, or Other ethnicity were associated with approximately 60% lower odds of quit success compared to White ethnicity patients at 6 months after discharge, a finding which was consistent across all our sensitivity analyses. This is despite a greater proportion accepting the intervention at first TDS assessment, reflecting international findings that diverse ethnic groups respond differently to smoking cessation interventions, and culturally tailored interventions may be required to improve outcomes [30, 31].

Conversely, patients with a history of diabetes were associated with 27% lower odds of accepting the intervention at first TDS assessment, but approximately 88% higher odds of reporting being a non-smoker 6 months after discharge. Previous research has identified a deterioration in glycaemic control that people with type 2 diabetes often experience after stopping smoking [32, 33], which could be a factor in explaining reticence to engage with smoking cessation interventions in this group.

Our study found that there was no difference in smoking cessation outcomes between those who were not prescribed smoking cessation aids and those who were, whether this was single or combination NRT. This is in contrast to the evidence from randomised trials among people motivated to quit, wherein NRT has been found to increase quit rates by 39% in trials initiated in a hospital setting [34]. However, observational data from clinical settings has shown the benefit of NRT on 4-week quit rates to be more marginal (49% quit with no NRT, compared to 56% with single NRT and 53% with combination NRT) [35], and previous research has found that the benefit of NRT as part of hospital-initiated tobacco dependence treatment is attenuated if follow-up is by phone rather than in-person, as was the case in our study [36]. Furthermore, as post-discharge NRT was provided by community services rather than the hospitals, we do not know the extent to which patients maintained their adherence to NRT after discharge.

Mental/behavioural disorders were highly prevalent, with almost two-thirds having ever had such a diagnosis and were associated with the greatest increase in odds of readmission and the second highest increase in odds of death (after cancer). The prevalence of mental illness among this sample is higher than in other published studies conducted on tobacco treatment interventions in similar settings [9, 37]. The finding that there was no difference in the uptake of support among these patients underscores that this is a population who want to quit [38]; however, as others have previously found [37, 39], the lower odds of successfully quitting—as found in our adjusted estimates at 90 days and in our unadjusted estimates at all time points—suggest there are challenges in turning this motivation into quit success.

### Strengths

Our pragmatic service evaluation reports real-world outcomes from a 1-year implementation of an opt-out smoking cessation intervention designed for hospital inpatient settings. The data come from two large central London teaching hospitals serving a diverse population, which includes many of the groups that suffer health inequalities and disproportionate harm from smoking.

Multiple imputation by chained equations was used to impute missing data—this is the ‘gold standard’ method for dealing with missing data which takes a principled approach and accounts for estimation error, whilst allowing the retention of all available information [40].

A range of sensitivity analyses were conducted to confirm the robustness of the results, which involved restricting the sample to patients with complete observed data, to the first admission of each patient only, and to only those patients who accepted the intervention at the first TDS assessment.

### Limitations

Data were collected from patients receiving care in London, UK, and thus may not be representative or generalisable to hospitals outside London. We were not able to compare the results to a control group as data were collected by the TDSs, and so, we do not have equivalent baseline or follow-up data from smokers who did not receive the intervention. The analysis is descriptive rather than predictive, and so, no causal interpretation can be ascribed to the interpretation of the covariate effects, which are limited to the *direct* effect of each covariate when others are held constant, rather than the *total* effect of any covariate (which would include mediation through other covariates).

As this was an observational study which did not involve randomisation, some residual confounding is inevitable. In particular, length of stay could not be included in the regression models due to violation of the assumption of the linearity of the logit. However, the length of stay was highly correlated with the hospital site, for which each model was adjusted. Similarly, although correlations between covariates were checked, some residual multicollinearity is inevitable, e.g. a moderate degree of association between tobacco dependence (as measured by HSI) and number of smoking cessation aids prescribed (Cramer’s *V* = 0.265), such that patients with more severe tobacco dependence were likely to be prescribed single or combination therapy, and which likely accounts for the finding that single pharmacotherapy had higher odds of readmission compared to those who were not prescribed smoking cessation aids.

Even after excluding codes relating to tobacco dependence, we used a very broad definition of mental and behavioural disorder, incorporating all conditions classified as such in the ICD-10, and a more granular analysis is warranted. Similarly, the sample size was not large enough to avoid collapsing certain sparse categories, especially with respect to the ethnicity category ‘Mixed/Asian/Others’.

Only 22% of the cohort were successfully followed up to 6 months, and smoking status was not biochemically verified. However, those patients who were not able to be contacted were conservatively classified as smokers, as per published guidance [41]. As NRT was provided by community services after discharge, we were also unable to verify if patients who were prescribed smoking cessation aids during admission continued to use them post-discharge.

### Future directions/implications

More research is needed to identify the elements of implementation that lead to optimal outcomes in hospital-initiated tobacco dependence treatment, such as how length of stay and the framing of offering tobacco dependence treatment during a hospital stay influence the uptake and subsequent outcomes. Promoting the use of a hospital admission as a teachable moment and springboard to address tobacco dependence in the long term may lead to better outcomes, rather than promoting an admission as an event that requires the short-term management of a period of temporary abstinence from tobacco smoking. A granular analysis of uptake and outcomes among patients with mental and behavioural disorders is needed in order to assess differential outcomes across different types of disorders, and further investigation of the role of ethnicity with more specific categories may inform culturally tailored interventions. Although our study assessed a range of physical health conditions, further analysis of how patterns of multimorbidity impact intervention acceptance and smoking cessation outcomes could enrich our understanding of how the OMSC can be best implemented.

## Conclusions

Our evaluation of an adapted Ottawa model of smoking cessation intervention implemented in two major acute London hospitals found the intervention was accepted by the majority who were offered it and resulted in 6-month quit rates comparable to international implementations of the Ottawa model. Outcomes varied according to patient age, ethnicity, severity of nicotine dependence, intention on admission, and present and past diagnoses and also varied across the two hospital sites, demonstrating the need for further research to develop the optimal implementation of inpatient tobacco dependence treatment during acute hospital admission.

### Supplementary Information

**Additional file 1:** **Table S1.** Sample characteristics: Ethnicity subgroups (*n*=2067). **Table S2.** Sample characteristics: Smoking cessation aids prescribed (primary). **Table S3.** Sample characteristics: Smoking cessation aids prescribed (secondary). **Table S4.** Summary statistics stratified by Accepted intervention at first TDS assessment. **Table S5.** Summary statistics stratified by Smoking status at 180 days. **Table S6.** Summary statistics stratified by All-cause death between 31 days and 1 year. **Table S7.** Summary statistics stratified by All-cause readmission between 31 days and 1 year. **Fig. S1.** Comparison of imputed values for Ethnicity, How-many-smoked, Time-to-first-cigarette, Borough, IMD decile, and Primary diagnosis. **Table S8.** Source data for regression, stratified by Hospital, outcome = Accepted intervention on first TDS assessment (*N*=2049). **Table S9.** Source data for regression, stratified by Hospital, outcome = Smoking status (Non-smoker) at 180 days post-discharge (*N*=1957). **Table S10.** Source data for regression, stratified by Hospital, outcome = Death by any cause 31 days to 1 year post-discharge (*N*=1770). **Table S11.** Source data for regression, stratified by Hospital, outcome = Readmission 31 days to 1-year post-discharge (N=1534). **Table S12.** Regression estimates for outcome Smoking status (non-smoker) at 30 days. **Table S13.** Regression estimates for outcome Smoking status (non-smoker) at 90 days. **Fig. S2.** Plot comparing estimates from primary and sensitivity regression models for outcome Accepted intervention at first TDS assessment. **Fig. S3.** Plot comparing estimates from primary and sensitivity regression models for outcome Smoking status (non-smoker) at 30 days. **Fig. S4.** Plot comparing estimates from primary and sensitivity regression models for outcome Smoking status (non-smoker) at 90 days. **Fig. S5.** Plot comparing estimates from primary and sensitivity regression models for outcome Smoking status (non-smoker) at 180 days. **Fig. S6.** Plot comparing estimates from primary and sensitivity regression models for outcome All-cause death between 31 days and 1-year. **Fig. S7.** Plot comparing estimates from primary and sensitivity regression models for outcome All-cause readmission between 31 days and 1-year.

## Data Availability

This study uses de-identified patient data and as such cannot be made available. For queries, please contact the corresponding author (john.robins@kcl.ac.uk).

## Abbreviations

AOR: Adjusted odds ratio
CI: Confidence interval
COPD: Chronic obstructive pulmonary disease
CURE: Conversation, Understand, Replace, Experts and evidence-based treatments
CVD: Cardiovascular disease
HSI: Heaviness of Smoking Index
ICD: International Classification of Diseases
IMD: Index of Multiple Deprivation
MUSC: Medical University of South Carolina
NHS: National Health Service
NRT: Nicotine replacement therapy
OMSC: Ottawa Model for Smoking Cessation
OR: Odds ratio
TDS: Tobacco dependence specialists
