# Supplementary material for: Evaluation of a hospital-initiated tobacco dependence treatment service: uptake, smoking cessation, readmission and mortality
Source: BMC Med. 2024 Mar 25;22:139. doi: 10.1186/s12916-024-03353-8 (PMC10964535; doi:10.1186/s12916-024-03353-8)
Supplement: Supplementary file 1 — Additional file 1: Table S1. Sample characteristics: Ethnicity subgroups (n=2067). Table S2. Sample characteristics: Smoking cessation aids prescribed (primary). Table S3. Sample characteristics: Smoking cessation aids prescribed (secondary). Table S4. Summary statistics stratified by Accepted intervention at first TDS assessment. Table S5. Summary statistics stratified by Smoking status at 180 days. Table S6. Summary statistics stratified by All-cause death between 31 days and 1 year. Table S7. Summary statistics stratified by All-cause readmission between 31 days and 1 year. Fig. S1. Comparison of imputed values for Ethnicity, How-many-smoked, Time-to-first-cigarette, Borough, IMD decile, and Primary diagnosis. Table S8. Source data for regression, stratified by Hospital, outcome = Accepted intervention on first TDS assessment (N=2049). Table S9. Source data for regression, stratified by Hospital, outcome = Smoking status (Non-smoker) at 180 days post-discharge (N=1957). Table S10. Source data for regression, stratified by Hospital, outcome = Death by any cause 31 days to 1 year post-discharge (N=1770). Table S11. Source data for regression, stratified by Hospital, outcome = Readmission 31 days to 1-year post-discharge (N=1534). Table S12. Regression estimates for outcome Smoking status (non-smoker) at 30 days. Table S13. Regression estimates for outcome Smoking status (non-smoker) at 90 days. Fig. S2. Plot comparing estimates from primary and sensitivity regression models for outcome Accepted intervention at first TDS assessment. Fig. S3. Plot comparing estimates from primary and sensitivity regression models for outcome Smoking status (non-smoker) at 30 days. Fig. S4. Plot comparing estimates from primary and sensitivity regression models for outcome Smoking status (non-smoker) at 90 days. Fig. S5. Plot comparing estimates from primary and sensitivity regression models for outcome Smoking status (non-smoker) at 180 days. Fig. S6. Plot comparing estimates from pr [file 12916_2024_3353_MOESM1_ESM.docx]

# Additional file

Contents

[Additional file 1](#_Toc160980894)

[Additional Table 1: Sample characteristics: *Ethnicity* subgroups (n=2067) 3](#_Toc160980895)

[Additional Table 2: Sample characteristics: *Smoking cessation aids prescribed* (primary) 4](#_Toc160980896)

[Additional Table 3: Sample characteristics: *Smoking cessation aids prescribed* (secondary) 4](#_Toc160980897)

[Summary statistics stratified by outcomes 5](#_Toc160980898)

[**Additional Table 4:** Summary statistics stratified by *Accepted intervention at first TDS assessment* 5](#_Toc160980899)

[**Additional Table 5:** Summary statistics stratified by *Smoking status at 180 days* 7](#_Toc160980900)

[**Additional Table 6:** Summary statistics stratified by *All-cause death between 31 days and 1 year* 10](#_Toc160980901)

[**Additional Table 7:** Summary statistics stratified by *All-cause readmission between 31 days and 1 year* 13](#_Toc160980902)

[Multiple imputation of missing data 16](#_Toc160980903)

[**Additional Figure 1:**  Comparison of imputed values for *Ethnicity*, *How-many-smoked*, *Time-to-first-cigarette*, *Borough*, *IMD decile*, and *Primary diagnosis*. 16](#_Toc160980904)

[Source data for regression analyses, stratified by Hospital and outcomes 17](#_Toc160980905)

[**Additional Table 8:** Source data for regression, stratified by *Hospital*, outcome = *Accepted intervention on first TDS assessment* (N=2049) 17](#_Toc160980906)

[**Additional Table 9:** Source data for regression, stratified by *Hospital*, outcome = *Smoking status (Non-smoker) at 180 days post-discharge* (N=1957) 19](#_Toc160980907)

[**Additional Table 10:** 21](#_Toc160980908)

[Source data for regression, stratified by *Hospital*, outcome = *Death by any cause 31 days to 1 year post-discharge* (N=1770) 21](#_Toc160980909)

[**Additional Table 11:** 23](#_Toc160980910)

[Source data for regression, stratified by *Hospital*, outcome = *Readmission 31 days to 1-year post-discharge* (N=1534) 23](#_Toc160980911)

[Additional Table 12: Regression estimates for outcome *Smoking status (non-smoker) at 30 days* 25](#_Toc160980912)

[Additional Table 13: Regression estimates for outcome *Smoking status (non-smoker) at 90 days* 26](#_Toc160980913)

[Sensitivity analyses: comparison of regression estimates from primary and sensitivity models 27](#_Toc160980914)

[**Additional Figure 2:** Plot comparing estimates from primary and sensitivity regression models for outcome *Accepted intervention at first TDS assessment* 27](#_Toc160980915)

[**Additional Figure 3:** Plot comparing estimates from primary and sensitivity regression models for outcome *Smoking status (non-smoker) at 30 days* 28](#_Toc160980916)

[**Additional Figure 4:** Plot comparing estimates from primary and sensitivity regression models for outcome *Smoking status (non-smoker) at 90 days* 29](#_Toc160980917)

[**Additional Figure 5:** Plot comparing estimates from primary and sensitivity regression models for outcome *Smoking status (non-smoker) at 180 days* 30](#_Toc160980918)

[**Additional Figure 6:** Plot comparing estimates from primary and sensitivity regression models for outcome *All-cause death between 31 days and 1-year* 31](#_Toc160980919)

[**Additional Figure 7:** Plot comparing estimates from primary and sensitivity regression models for outcome *All-cause readmission between 31 days and 1-year* 32](#_Toc160980920)

## **Additional Table 1:** Sample characteristics: *Ethnicity* subgroups (n=2067)

| Ethnicity category | N | % |
| --- | --- | --- |
| Asian: Any other | 18 | 0.9 |
| Asian: Bangladeshi | 21 | 1 |
| Asian: Chinese | <10 | 0.4 |
| Asian: Indian | <10 | 0.4 |
| Asian: Pakistani | <10 | 0.2 |
| Black: African | 31 | 1.5 |
| Black: Any other | 103 | 5 |
| Black: Caribbean | 99 | 4.8 |
| Black: Unspecified | <10 | 0.2 |
| Mixed: Any other | 10 | 0.5 |
| Mixed: White and Asian | <10 | 0 |
| Mixed: White and Black African | <10 | 0.3 |
| Mixed: White and Black Caribbean | 25 | 1.2 |
| White: Any other | 198 | 9.6 |
| White: British | 1051 | 50.8 |
| White: Irish | 54 | 2.6 |
| White: Unspecified | <10 | 0.1 |
| Other: Any other | 117 | 5.7 |
| Unknown / declined | 234 | 11.3 |
| Missing | 68 | 3.3 |

Frequencies fewer than 10 suppressed to prevent deanonymisation

## **Additional Table 2:** Sample characteristics: *Smoking cessation aids prescribed* (primary)

| Product | N | % |
| --- | --- | --- |
| Patch 20mg+ | 642 | *31.1* |
| Inhalator 15mg | 241 | *11.7* |
| Patch <20mg | 176 | *8.5* |
| Mouth Spray | 63 | *3* |
| Varenicline | 15 | *0.7* |
| Minis | 14 | *0.7* |
| Gum | 12 | *0.6* |
| Lozenges | <10 | *0.3* |
| No medication 1 recorded | 898 | *43.4* |

Frequencies fewer than 10 suppressed to prevent deanonymisation

## **Additional Table 3:** Sample characteristics: *Smoking cessation aids prescribed* (secondary)

| Product | N | % |
| --- | --- | --- |
| Inhalator | 455 | *22* |
| Mouth Spray | 124 | *6* |
| Patches (strength unspecified) | 47 | *2.3* |
| Gum | 32 | *1.5* |
| Lozenges | 25 | *1.2* |
| Minis | 16 | *0.8* |
| Varenicline | <10 | *0.1* |
| No medication 2 recorded | 1365 | *66* |

Frequencies fewer than 10 suppressed to prevent deanonymisation

## Summary statistics stratified by outcomes

### **Additional Table 4:** Summary statistics stratified by *Accepted intervention at first TDS assessment*

|  |  | **N=2067** | | **Outcome:**  **Accepted intervention at first TDS assessment** | | | | |
| --- | --- | --- | --- | --- | --- | --- | --- | --- |
| **Variable** | **Categories** | **Total N** | **Total %** | **Yes n** | **Yes %** | **No n** | **No %** | **p-value** |
| **Hospital** | A | 1394 | *67.4* | 1047 | *63.8* | 340 | *83.5* | <0.001 |
|  | B | 673 | *32.6* | 595 | *36.2* | 67 | *16.5* |  |
| **Age** | 16-24 | 126 | *6.1* | 89 | *5.4* | 36 | *8.8* | 0.045 |
|  | 25-39 | 427 | *20.7* | 334 | *20.3* | 87 | *21.4* |  |
|  | 40-59 | 784 | *37.9* | 629 | *38.3* | 151 | *37.1* |  |
|  | 60+ | 679 | *32.8* | 551 | *33.6* | 121 | *29.7* |  |
|  | *Missing* | *51* | *2.5* | *39* | *2.4* | *12* | *2.9* |  |
| **Sex** | Male | 1334 | *64.5* | 1055 | *64.3* | 266 | *65.4* | 0.608 |
|  | Female | 723 | *35.0* | 581 | *35.4* | 137 | *33.7* |  |
|  | *Missing* | *10* | *0.5* | *<10* | *-* | *<10* | *-* |  |
| **Ethnicity** | Asian | 61 | *3.0* | 49 | *3.0* | 12 | *2.9* | 0.039 |
|  | Black | 238 | *11.5* | 204 | *12.4* | 31 | *7.6* |  |
|  | White | 1306 | *63.2* | 1019 | *62.1* | 278 | *68.3* |  |
|  | Mixed | 43 | *2.1* | 35 | *2.1* | <10 | *-* |  |
|  | Other | 117 | *5.7* | 99 | *6.0* | 16 | *3.9* |  |
|  | Declined / Not stated | 234 | *11.3* | 183 | *11.1* | 51 | *12.5* |  |
|  | *Missing* | *68* | *3.3* | *53* | *3.2* | *11* | *2.7* |  |
| **HSI category** | Low | 348 | *16.8* | 319 | *19.4* | 27 | *6.6* | 0.702 |
|  | Medium | 774 | *37.4* | 699 | *42.6* | 72 | *17.7* |  |
|  | High | 108 | *5.2* | 96 | *5.8* | <10 | *-* |  |
|  | *Missing* | *837* | *40.5* | *528* | *32.2* | *299* | *73.5* |  |
| **Smoking related prim. dx.** | Yes | 399 | *19.3* | 339 | *20.6* | 59 | *14.5* | 0.053 |
|  | No | 1257 | *60.8* | 1004 | *61.1* | 240 | *59.0* |  |
|  | *Missing* | *411* | *19.9* | *299* | *18.2* | *108* | *26.5* |  |
| **Present. primary diagnosis** | Injury / poison. / ext. | 306 | *14.8* | 255 | *15.5* | 43 | *10.6* | 0.008 |
|  | Digestive | 215 | *10.4* | 178 | *10.8* | 36 | *8.8* |  |
|  | Mental & behav. | 214 | *10.4* | 163 | *9.9* | 49 | *12.0* |  |
|  | Respiratory | 205 | *9.9* | 177 | *10.8* | 28 | *6.9* |  |
|  | Circulatory | 131 | *6.3* | 110 | *6.7* | 21 | *5.2* |  |
|  | Endocrine | 106 | *5.1* | 85 | *5.2* | 21 | *5.2* |  |
|  | Genitourinary | 64 | *3.1* | 53 | *3.2* | 11 | *2.7* |  |
|  | Neoplasms | 63 | *3.0* | 52 | *3.2* | 10 | *2.5* |  |
|  | Infectious | 49 | *2.4* | 44 | *2.7* | <10 | *-* |  |
|  | Musculoskeletal | 47 | *2.3* | 36 | *2.2* | 10 | *2.5* |  |
|  | Nervous system | 46 | *2.2* | 37 | *2.3* | <10 | *-* |  |
|  | Skin / subcutaneous | 37 | *1.8* | 23 | *1.4* | 14 | *3.4* |  |
|  | Other | 173 | *8.4* | 130 | *7.9* | 42 | *10.3* |  |
|  | *Missing* | *411* | *19.9* | *299* | *18.2* | *108* | *26.5* |  |
| **Past diagnoses** | Cancer | 217 | *10.5* | 170 | *10.4* | 47 | *11.5* | 0.541 |
|  | COPD | 557 | *26.9* | 456 | *27.8* | 98 | *24.1* | 0.150 |
|  | CVD | 310 | *15.0* | 229 | *13.9* | 79 | *19.4* | 0.007 |
|  | Diabetes | 438 | *21.2* | 325 | *19.8* | 110 | *27.0* | 0.002 |
|  | Mental & behav. | 1375 | *66.5* | 1081 | *65.8* | 281 | *69.0* | 0.243 |
| **IMD tertile** | Lower | 855 | *41.4* | 704 | *42.9* | 144 | *35.4* | 0.006 |
|  | Middle | 509 | *24.6* | 409 | *24.9* | 96 | *23.6* |  |
|  | Upper | 133 | *6.4* | 102 | *6.2* | 29 | *7.1* |  |
|  | *Missing* | *570* | *27.6* | *427* | *26.0* | *138* | *33.9* |  |

IMD = Index of Multiple Deprivation (only available for patients resident in England), HSI = Heaviness of Smoking Index, TDS = Tobacco Dependence Specialists

Frequencies fewer than 10 supressed to prevent potential de-anonymisation

### **Additional Table 5:** Summary statistics stratified by *Smoking status at 180 days*

|  |  | **N=1957*** | | **Outcome: Smoking status at 180 days** | | | | |
| --- | --- | --- | --- | --- | --- | --- | --- | --- |
|  |  |  |  | **Smoker / unknown** | | **Non-smoker** | |  |
| **Variable** | **Categories** | **Total N** | **Total %** | **n** | **%** | **n** | **%** | **p-value** |
| **Hospital** | A | 1306 | 66.7 | 1258 | 69.7 | 48 | 31.6 | <0.001 |
|  | B | 651 | 33.3 | 547 | 30.3 | 104 | 68.4 |  |
| **Age** | 16-24 | 125 | 6.4 | 120 | 6.6 | <10 | - | 0.004 |
|  | 25-39 | 421 | 21.5 | 392 | 21.7 | 29 | 19.1 |  |
|  | 40-59 | 757 | 38.7 | 707 | 39.2 | 50 | 32.9 |  |
|  | 60+ | 608 | 31.1 | 541 | 30.0 | 67 | 44.1 |  |
|  | *Missing* | 46 | 2.4 | 45 | 2.5 | <10 | - |  |
| **Sex** | Male | 1254 | 64.1 | 1158 | 64.2 | 96 | 63.2 | 0.805 |
|  | Female | 693 | 35.4 | 637 | 35.3 | 56 | 36.8 |  |
|  | *Missing* | 10 | 0.5 | 10 | 0.6 | <10 | - |  |
| **Ethnicity** | Asian | 58 | 3.0 | 56 | 3.1 | <10 | - | 0.008 |
|  | Black | 231 | 11.8 | 201 | 11.1 | 30 | 19.7 |  |
|  | White | 1219 | 62.3 | 1121 | 62.1 | 98 | 64.5 |  |
|  | Mixed | 43 | 2.2 | 42 | 2.3 | <10 | - |  |
|  | Other | 114 | 5.8 | 108 | 6.0 | <10 | - |  |
|  | Declined / Not stated | 226 | 11.5 | 215 | 11.9 | 11 | 7.2 |  |
|  | *Missing* | 66 | 3.4 | 62 | 3.4 | <10 | - |  |
| **Intention at first TDS assessment** | Pt declined | 377 | 19.3 | 372 | 20.6 | <10 | - | <0.001 |
|  | Withdrawal mgmt. | 1040 | 53.1 | 988 | 54.7 | 52 | 34.2 |  |
|  | Quit attempt | 523 | 26.7 | 431 | 23.9 | 92 | 60.5 |  |
|  | *Missing* | 17 | 0.9 | 14 | 0.8 | <10 | - |  |
| **HSI category** | Low | 338 | 17.3 | 278 | 15.4 | 60 | 39.5 | <0.001 |
|  | Medium | 742 | 37.9 | 690 | 38.2 | 52 | 34.2 |  |
|  | High | 104 | 5.3 | 98 | 5.4 | <10 | - |  |
|  | *Missing* | 773 | 39.5 | 739 | 40.9 | 34 | 22.4 |  |
| **Smoking cessation aids** | None | 838 | 42.8 | 763 | 42.3 | 75 | 49.3 | 0.177 |
|  | Single | 441 | 22.5 | 409 | 22.7 | 32 | 21.1 |  |
|  | Combination | 668 | 34.1 | 625 | 34.6 | 43 | 28.3 |  |
|  | *Missing* | 10 | 0.5 | <10 | - | <10 | - |  |
| **Smoking related prim. dx.** | Yes | 354 | 18.1 | 305 | 16.9 | 49 | 32.2 | <0.001 |
|  | No | 1210 | 61.8 | 1122 | 62.2 | 88 | 57.9 |  |
|  | *Missing* | 393 | 20.1 | 378 | 20.9 | 15 | 9.9 |  |
| **Present. primary diagnosis** | Injury / poison. / ext. | 119 | 6.1 | 98 | 5.4 | 21 | 13.8 | <0.001 |
|  | Digestive | 207 | 10.6 | 190 | 10.5 | 17 | 11.2 |  |
|  | Mental & behav. | 99 | 5.1 | 89 | 4.9 | 10 | 6.6 |  |
|  | Respiratory | 63 | 3.2 | 56 | 3.1 | <10 | - |  |
|  | Circulatory | 47 | 2.4 | 40 | 2.2 | <10 | - |  |
|  | Endocrine | 301 | 15.4 | 270 | 15.0 | 31 | 20.4 |  |
|  | Genitourinary | 203 | 10.4 | 200 | 11.1 | <10 | - |  |
|  | Neoplasms | 45 | 2.3 | 41 | 2.3 | <10 | - |  |
|  | Infectious | 47 | 2.4 | 37 | 2.0 | 10 | 6.6 |  |
|  | Musculoskeletal | 43 | 2.2 | 40 | 2.2 | <10 | - |  |
|  | Nervous system | 188 | 9.6 | 170 | 9.4 | 18 | 11.8 |  |
|  | Skin / subcutaneous | 36 | 1.8 | 36 | 2.0 | <10 | - |  |
|  | Other | 166 | 8.5 | 160 | 8.9 | <10 | - |  |
|  | *Missing* | 393 | 20.1 | 378 | 20.9 | 15 | 9.9 |  |
| **Past diagnoses** | Cancer | 169 | 8.6 | 159 | 8.8 | 10 | 6.6 | 0.430 |
|  | COPD | 498 | 25.4 | 455 | 25.2 | 43 | 28.3 | 0.459 |
|  | CVD | 275 | 14.1 | 246 | 13.6 | 29 | 19.1 | 0.083 |
|  | Diabetes | 386 | 19.7 | 350 | 19.4 | 36 | 23.7 | 0.241 |
|  | Mental & behav. | 1297 | 66.3 | 1223 | 67.8 | 74 | 48.7 | <0.001 |
| **IMD tertile** | Lower | 795 | 40.6 | 722 | 40.0 | 73 | 48.0 | 0.010 |
|  | Middle | 481 | 24.6 | 438 | 24.3 | 43 | 28.3 |  |
|  | Upper | 129 | 6.6 | 118 | 6.5 | 11 | 7.2 |  |
|  | *Missing* | 552 | 28.2 | 527 | 29.2 | 25 | 16.4 |  |

* Patient deaths within 180 days removed

IMD = Index of Multiple Deprivation (only available for patients resident in England), HSI = Heaviness of Smoking Index, TDS = Tobacco Dependence Specialists

Frequencies fewer than 10 supressed to prevent potential de-anonymisation

### **Additional Table 6:** Summary statistics stratified by *All-cause death between 31 days and 1 year*

|  |  | **First admission only (N=1770)** | | **Outcome:**  **All-cause death between 31 days to 1-year post-discharge*** | | | | |
| --- | --- | --- | --- | --- | --- | --- | --- | --- |
| **Variable** | **Categories** | **Total N** | **Total %** | **Yes n** | **Yes %** | **No n** | **No %** | **p-value** |
| **Hospital** | A | 1125 | *63.6* | 79 | *72.5* | 1046 | *63.0* | 0.058 |
|  | B | 645 | *36.4* | 30 | *27.5* | 615 | *37.0* |  |
| **Age** | 16-24 | 115 | *6.5* | 0 | *0* | 115 | *6.9* | <0.001 |
|  | 25-39 | 378 | *21.4* | <10 | *-* | 375 | *22.6* |  |
|  | 40-59 | 683 | *38.6* | 34 | *31.2* | 649 | *39.1* |  |
|  | 60+ | 554 | *31.3* | 69 | *63.3* | 485 | *29.2* |  |
|  | *Missing* | *40* | *2.3* | *<10* | *-* | *37* | *2.2* |  |
| **Sex** | Male | 1128 | *63.7* | 82 | *75.2* | 1046 | *63.0* | 0.016 |
|  | Female | 632 | *35.7* | 27 | *24.8* | 605 | *36.4* |  |
|  | *Missing* | *10* | *0.6* | *0* | *0* | *10* | *0.6* |  |
| **Ethnicity** | Asian | 43 | *2.4* | <10 | *-* | 41 | *2.5* | 0.105 |
|  | Black | 214 | *12.1* | <10 | *-* | 206 | *12.4* |  |
|  | White | 1086 | *61.4* | 82 | *75.2* | 1004 | *60.4* |  |
|  | Mixed | 41 | *2.3* | 0 | *0* | 41 | *2.5* |  |
|  | Other | 114 | *6.4* | <10 | *-* | 107 | *6.4* |  |
|  | Declined / Not stated | 215 | *12.1* | 10 | *9.2* | 205 | *12.3* |  |
|  | *Missing* | *57* | *3.2* | *0* | *0* | *57* | *3.4* |  |
| **HSI category** | Low | 316 | *17.9* | 11 | *10.1* | 305 | *18.4* | 0.027 |
|  | Medium | 655 | *37* | 38 | *34.9* | 617 | *37.1* |  |
|  | High | 88 | *5* | 0 | *0* | 88 | *5.3* |  |
|  | *Missing* | *711* | *40.2* | *60* | *55.0* | *651* | *39.2* |  |
| **Smoking related prim. dx.** | Yes | 334 | *18.9* | 47 | *43.1* | 287 | *17.3* | <0.001 |
|  | No | 1089 | *61.5* | 57 | *52.3* | 1032 | *62.1* |  |
|  | *Missing* | *347* | *19.6* | *<10* | *-* | *342* | *20.6* |  |
| **Present. primary diagnosis** | Injury / poison. / ext. | 111 | *6.3* | 16 | *14.7* | 95 | *5.7* | <0.001 |
|  | Digestive | 176 | *9.9* | <10 | *-* | 168 | *10.1* |  |
|  | Mental & behav. | 94 | *5.3* | 10 | *9.2* | 84 | *5.1* |  |
|  | Respiratory | 59 | *3.3* | <10 | *-* | 56 | *3.4* |  |
|  | Circulatory | 47 | *2.7* | <10 | *-* | 44 | *2.6* |  |
|  | Endocrine | 286 | *16.2* | <10 | *-* | 278 | *16.7* |  |
|  | Genitourinary | 175 | *9.9* | <10 | *-* | 169 | *10.2* |  |
|  | Neoplasms | 43 | *2.4* | <10 | *-* | 38 | *2.3* |  |
|  | Infectious | 51 | *2.9* | 16 | *14.7* | 35 | *2.1* |  |
|  | Musculoskeletal | 38 | *2.1* | <10 | *-* | 36 | *2.2* |  |
|  | Nervous system | 172 | *9.7* | 15 | *13.8* | 157 | *9.5* |  |
|  | Skin / subcutaneous | 32 | *1.8* | <10 | *-* | 31 | *1.9* |  |
|  | Other | 139 | *7.9* | 11 | *10.1* | 128 | *7.7* |  |
|  | *Missing* | *347* | *19.6* | *<10* | *-* | *342* | *20.6* |  |
| **Past diagnoses** | Cancer | 170 | *9.6* | 40 | *36.7* | 130 | *7.8* | <0.001 |
|  | COPD | 421 | *23.8* | 48 | *44.0* | 373 | *22.5* | <0.001 |
|  | CVD | 212 | *12* | 30 | *27.5* | 182 | *11.0* | <0.001 |
|  | Diabetes | 316 | *17.9* | 42 | *38.5* | 274 | *16.5* | <0.001 |
|  | Mental & behav. | 1122 | *63.4* | 81 | *74.3* | 1041 | *62.7* | 0.019 |
| **IMD tertile** | Lower | 727 | *41.1* | 65 | *59.6* | 662 | *39.9* | <0.001 |
|  | Middle | 429 | *24.2* | 28 | *25.7* | 401 | *24.1* |  |
|  | Upper | 120 | *6.8* | <10 | *-* | 116 | *7.0* |  |
|  | *Missing* | *494* | *27.9* | *12* | *11* | *482* | *29* |  |
| **Intention at first TDS assessment .** | Pt declined | 336 | *19* | 28 | *25.7* | 308 | *18.5* | 0.007 |
|  | Withdrawal mgmt. | 923 | *52.1* | 64 | *58.7* | 859 | *51.7* |  |
|  | Quit attempt | 495 | *28* | 17 | *15.6* | 478 | *28.8* |  |
|  | *Missing* | *16* | *0.9* | *0* | *0* | *16* | *1.0* |  |
| **Smoking cessation aids** | None | 758 | *42.8* | 48 | *44.0* | 710 | *42.7* | 0.893 |
|  | Single | 404 | *22.8* | 23 | *21.1* | 381 | *22.9* |  |
|  | Combination | 598 | *33.8* | 38 | *34.9* | 560 | *33.7* |  |
|  | *Missing* | *10* | *0.6* | *0* | *0* | *10* | *0.6* |  |
| **Smoking status 30 days** | Non-Smoker | 171 | *9.7* | 10 | *9.2* | 161 | *9.7* | 0.833 |
|  | Smoker | 377 | *21.3* | 24 | *22.0* | 353 | *21.3* |  |
|  | Unknown | 926 | *52.3* | 51 | *46.8* | 875 | *52.7* |  |
|  | *Missing* | *296* | *16.7* | *24* | *22.0* | *272* | *16.4* |  |

* Patient deaths within 30 days removed

IMD = Index of Multiple Deprivation (only available for patients resident in England), HSI = Heaviness of Smoking Index, TDS = Tobacco Dependence Specialists

Frequencies fewer than 10 supressed to prevent potential de-anonymisation

### **Additional Table 7:** Summary statistics stratified by *All-cause readmission between 31 days and 1 year*

|  |  | **N=2030*** | | **Outcome:**  **All-cause readmission between 31 days to 1-year post-discharge** | | | | |
| --- | --- | --- | --- | --- | --- | --- | --- | --- |
| **Variable** | **Categories** | **Total N** | **Total %** | **Yes n** | **Yes %** | **No n** | **No %** | **p-value** |
| **Hospital** | A | 1371 | 67.5 | 352 | 76.2 | 1019 | 65.0 | <0.001 |
|  | B | 659 | 32.5 | 110 | 23.8 | 549 | 35.0 |  |
| **Age** | 16-24 | 125 | 6.2 | 16 | 3.5 | 109 | 7.0 | <0.001 |
|  | 25-39 | 424 | 20.9 | 57 | 12.3 | 367 | 23.4 |  |
|  | 40-59 | 776 | 38.2 | 203 | 43.9 | 573 | 36.5 |  |
|  | 60+ | 656 | 32.3 | 181 | 39.2 | 475 | 30.3 |  |
|  | *Missing* | 49 | 2.4 | *<10* | *-* | *44* | *2.8* |  |
| **Sex** | Male | 1309 | 64.5 | 288 | 62.3 | 1021 | 65.1 | 0.227 |
|  | Female | 711 | 35 | 174 | 37.7 | 537 | 34.2 |  |
|  | *Missing* | 10 | 0.5 | *0* | *0.0* | *10* | *0.6* |  |
| **Ethnicity** | Asian | 60 | 3 | 15 | 3.2 | 45 | 2.9 | 0.029 |
|  | Black | 235 | 11.6 | 54 | 11.7 | 181 | 11.5 |  |
|  | White | 1278 | 63 | 322 | 69.7 | 956 | 61.0 |  |
|  | Mixed | 43 | 2.1 | 13 | 2.8 | 30 | 1.9 |  |
|  | Other | 116 | 5.7 | 21 | 4.5 | 95 | 6.1 |  |
|  | Declined / Not stated | 232 | 11.4 | 37 | 8.0 | 195 | 12.4 |  |
|  | *Missing* | 66 | 3.3 | *0* | *0.0* | *66* | *4.2* |  |
| **HSI category** | Low | 344 | 16.9 | 72 | 15.6 | 272 | 17.3 | 0.539 |
|  | Medium | 762 | 37.5 | 181 | 39.2 | 581 | 37.1 |  |
|  | High | 104 | 5.1 | 22 | 4.8 | 82 | 5.2 |  |
|  | *Missing* | 820 | 40.4 | *187* | *40.5* | *633* | *40.4* |  |
| **Smoking related prim. dx.** | Yes | 388 | 19.1 | 104 | 22.5 | 284 | 18.1 | 0.128 |
|  | No | 1242 | 61.2 | 284 | 61.5 | 958 | 61.1 |  |
|  | *Missing* | 400 | 19.7 | *74* | *16.0* | *326* | *20.8* |  |
| **Present. primary diagnosis** | Injury / poison. / ext. | 129 | 6.4 | 34 | 7.4 | 95 | 6.1 | <0.001 |
|  | Digestive | 211 | 10.4 | 70 | 15.2 | 141 | 9.0 |  |
|  | Mental & behav. | 104 | 5.1 | 27 | 5.8 | 77 | 4.9 |  |
|  | Respiratory | 64 | 3.2 | 13 | 2.8 | 51 | 3.3 |  |
|  | Circulatory | 49 | 2.4 | 15 | 3.2 | 34 | 2.2 |  |
|  | Endocrine | 305 | 15 | 40 | 8.7 | 265 | 16.9 |  |
|  | Genitourinary | 210 | 10.3 | 48 | 10.4 | 162 | 10.3 |  |
|  | Neoplasms | 47 | 2.3 | 11 | 2.4 | 36 | 2.3 |  |
|  | Infectious | 58 | 2.9 | 10 | 2.2 | 48 | 3.1 |  |
|  | Musculoskeletal | 45 | 2.2 | 15 | 3.2 | 30 | 1.9 |  |
|  | Nervous system | 201 | 9.9 | 60 | 13.0 | 141 | 9.0 |  |
|  | Skin / subcutaneous | 37 | 1.8 | *<10* | *-* | 34 | 2.2 |  |
|  | Other | 170 | 8.4 | 42 | 9.1 | 128 | 8.2 |  |
|  | *Missing* | 400 | 19.7 | *74* | *16.0* | *326* | *20.8* |  |
| **Previous diagnoses** | Cancer | 204 | 10 | 74 | 16.0 | 130 | 8.3 | <0.001 |
|  | COPD | 542 | 26.7 | 194 | 42.0 | 348 | 22.2 | <0.001 |
|  | CVD | 299 | 14.7 | 95 | 20.6 | 204 | 13.0 | <0.001 |
|  | Diabetes | 427 | 21 | 133 | 28.8 | 294 | 18.8 | <0.001 |
|  | Mental & behav. | 1352 | 66.6 | 378 | 81.8 | 974 | 62.1 | <0.001 |
| **IMD tertile** | Lower | 836 | 41.2 | 233 | 50.4 | 603 | 38.5 | <0.001 |
|  | Middle | 501 | 24.7 | 112 | 24.2 | 389 | 24.8 |  |
|  | Upper | 131 | 6.5 | 25 | 5.4 | 106 | 6.8 |  |
|  | *Missing* | 562 | 27.7 | *92* | *19.9* | *470* | *30.0* |  |
| **Intention at first TDS assessment** | Pt declined | 401 | 19.8 | 91 | 19.7 | 310 | 19.8 | 0.113 |
|  | Withdrawal mgmt. | 1078 | 53.1 | 264 | 57.1 | 814 | 51.9 |  |
|  | Quit attempt | 534 | 26.3 | 106 | 22.9 | 428 | 27.3 |  |
|  | *Missing* | 17 | 0.8 | *<10* | *-* | *16* | *1.0* |  |
| **Smoking cessation aids** | None | 872 | 43 | 167 | 36.1 | 705 | 45.0 | 0.002 |
|  | Single | 455 | 22.4 | 113 | 24.5 | 342 | 21.8 |  |
|  | Combination | 693 | 34.1 | 181 | 39.2 | 512 | 32.7 |  |
|  | *Missing* | 10 | 0.5 | *<10* | *-* | *<10* | *-* |  |
| **Smoking status 30 days** | Non-Smoker | 195 | 9.6 | 36 | 7.8 | 159 | 10.1 | 0.129 |
|  | Smoker | 406 | 20 | 104 | 22.5 | 302 | 19.3 |  |
|  | Unknown | 1070 | 52.7 | 238 | 51.5 | 832 | 53.1 |  |
|  | *Missing* | 359 | 17.7 | *84* | *18.2* | *275* | *17.5* |  |

* Patient deaths within 30 days removed

IMD = Index of Multiple Deprivation (only available for patients resident in England), HSI = Heaviness of Smoking Index, TDS = Tobacco Dependence Specialists

Frequencies fewer than 10 supressed to prevent potential de-anonymisation

## Multiple imputation of missing data

### **Additional Figure 1:** Comparison of imputed values for *Ethnicity*, *How-many-smoked*, *Time-to-first-cigarette*, *Borough*, *IMD decile*, and *Primary diagnosis*.


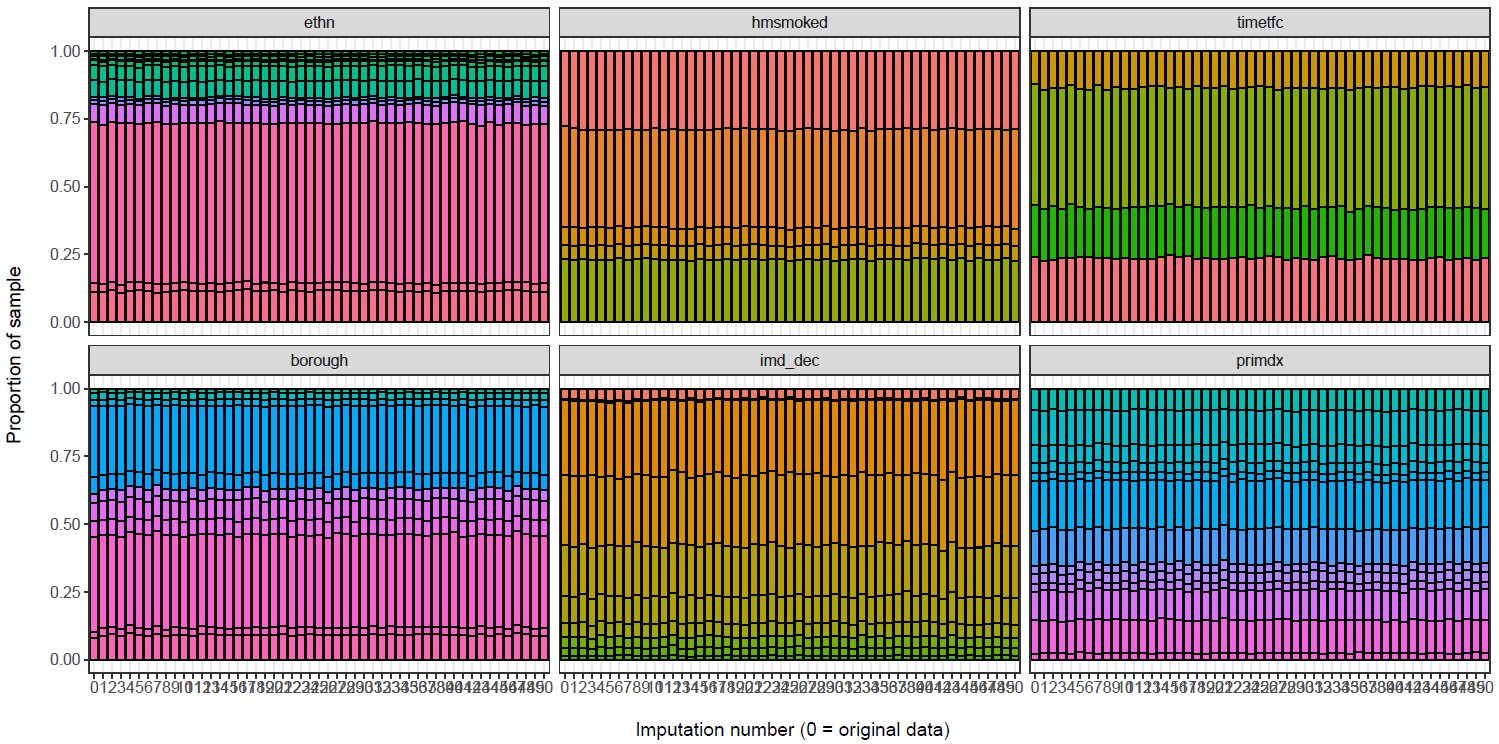


In each plot, the far left column represents the original data, with columns to the right representing each of the 50 imputed datasets. For each variable, there is some variation in the imputed values but the overall picture is consistent

## Source data for regression analyses, stratified by Hospital and outcomes

### **Additional Table 8:** Source data for regression, stratified by *Hospital*, outcome = *Accepted intervention on first TDS assessment* (N=2049)

|  |  | **Hospital A** (N=1387)^1^ | | | |  | **Hospital B** (N=662)^1^ | | | |  |
| --- | --- | --- | --- | --- | --- | --- | --- | --- | --- | --- | --- |
|  |  | **Accepted intervention** | | | |  | **Accepted intervention** | | | |  |
|  |  | ***No***  *(n=340, 24.5%)* | | ***Yes***  *(n=1047, 75.5%)* | |  | ***No***  *(n=67, 10.1%)* | | ***Yes***  *(n=595, 89.9%)* | |  |
| **Variable** | **Categories** | **n** | ***%*** | **n** | ***%*** | **p-value** | **n** | ***%*** | **n** | ***%*** | **p-value** |
| **Age On Admission** | 16-24 | 29 | ***8.5*** | 55 | ***5.3*** | 0.144 | <10 | ***-*** | 34 | ***5.7*** | 0.013 |
|  | 25-39 | 70 | ***20.6*** | 210 | ***20.1*** |  | 17 | ***25.4*** | 124 | ***20.8*** |  |
|  | 40-59 | 119 | ***35*** | 403 | ***38.5*** |  | 32 | ***47.8*** | 226 | ***38*** |  |
|  | 60+ | 110 | ***32.4*** | 340 | ***32.5*** |  | 11 | ***16.4*** | 211 | ***35.5*** |  |
|  | *Missing* | *12* | ***3.5*** | *39* | ***3.7*** |  | *<10* | ***-*** | *<10* | ***-*** |  |
| **Sex** | Male | 224 | ***65.9*** | 683 | ***65.2*** | 0.773 | 42 | ***62.7*** | 372 | ***62.5*** | 1.000 |
|  | Female | 112 | ***32.9*** | 358 | ***34.2*** |  | 25 | ***37.3*** | 223 | ***37.5*** |  |
|  | *Missing* | *<10* | ***-*** | *<10* | ***-*** |  | *<10* | ***-*** | *<10* | ***-*** |  |
| **Ethnicity** | Black | 25 | ***7.4*** | 88 | ***8.4*** | 0.871 | <10 | ***-*** | 116 | ***19.5*** | 0.071 |
|  | Mixed/Asian/Other^2^ | 29 | ***8.5*** | 93 | ***8.9*** |  | <10 | ***-*** | 90 | ***15.1*** |  |
|  | White | 233 | ***68.5*** | 683 | ***65.2*** |  | 45 | ***67.2*** | 336 | ***56.5*** |  |
|  | Declined or Not stated | 42 | ***12.4*** | 130 | ***12.4*** |  | <10 | ***-*** | 53 | ***8.9*** |  |
|  | *Missing* | *11* | ***3.2*** | *53* | ***5.1*** |  | *<10* | ***-*** | *<10* | ***-*** |  |
| **HSI Category** | Low | 15 | ***4.4*** | 139 | ***13.3*** | 0.616 | 12 | ***17.9*** | 180 | ***30.3*** | 0.340 |
|  | Medium | 47 | ***13.8*** | 433 | ***41.4*** |  | 25 | ***37.3*** | 266 | ***44.7*** |  |
|  | High | <10 | ***-*** | 62 | ***5.9*** |  | <10 | ***-*** | 34 | ***5.7*** |  |
|  | *Missing* | *274* | ***80.6*** | *413* | ***39.4*** |  | *25* | ***37.3*** | *115* | ***19.3*** |  |
| **Smoking related diagnosis** | Yes | 46 | ***13.5*** | 200 | ***19.1*** | 0.066 | 13 | ***19.4*** | 139 | ***23.4*** | 0.633 |
|  | No | 192 | ***56.5*** | 587 | ***56.1*** |  | 48 | ***71.6*** | 417 | ***70.1*** |  |
|  | *Missing* | *102* | ***30*** | *260* | ***24.8*** |  | *<10* | ***-*** | *39* | ***6.6*** |  |
| **Cancer** | Yes | 45 | ***13.2*** | 134 | ***12.8*** | 0.908 | <10 | ***-*** | 36 | ***6.1*** | 0.456 |
|  | No | 295 | ***86.8*** | 913 | ***87.2*** |  | 65 | ***97*** | 559 | ***93.9*** |  |
| **COPD** | Yes | 81 | ***23.8*** | 320 | ***30.6*** | 0.021 | 17 | ***25.4*** | 136 | ***22.9*** | 0.756 |
|  | No | 259 | ***76.2*** | 727 | ***69.4*** |  | 50 | ***74.6*** | 459 | ***77.1*** |  |
| **CVD** | Yes | 69 | ***20.3*** | 165 | ***15.8*** | 0.063 | 10 | ***14.9*** | 64 | ***10.8*** | 0.411 |
|  | No | 271 | ***79.7*** | 882 | ***84.2*** |  | 57 | ***85.1*** | 531 | ***89.2*** |  |
| **Diabetes** | Yes | 102 | ***30*** | 264 | ***25.2*** | 0.095 | <10 | ***-*** | 61 | ***10.3*** | 0.828 |
|  | No | 238 | ***70*** | 783 | ***74.8*** |  | 59 | ***88.1*** | 534 | ***89.7*** |  |
| **Mental Illness** | Yes | 247 | ***72.6*** | 786 | ***75.1*** | 0.413 | 34 | ***50.7*** | 295 | ***49.6*** | 0.958 |
|  | No | 93 | ***27.4*** | 261 | ***24.9*** |  | 33 | ***49.3*** | 300 | ***50.4*** |  |
| **IMD tertile** | Lower | 107 | ***31.5*** | 418 | ***39.9*** | 0.004 | 37 | ***55.2*** | 286 | ***48.1*** | 0.311 |
|  | Middle | 77 | ***22.6*** | 239 | ***22.8*** |  | 19 | ***28.4*** | 170 | ***28.6*** |  |
|  | Upper | 27 | ***7.9*** | 45 | ***4.3*** |  | <10 | ***-*** | 57 | ***9.6*** |  |
|  | *Missing* | *129* | ***37.9*** | *345* | ***33*** |  | *<10* | ***-*** | *82* | ***13.8*** |  |

^1^ *18 observations with missing outcome data not shown ^2^ Categories collapsed due to sparse data*

### **Additional Table 9:** Source data for regression, stratified by *Hospital*, outcome = *Smoking status (Non-smoker) at 180 days post-discharge* (N=1957)

|  |  | **Hospital A** (N=1306)^1^ | | | |  | **Hospital B** (N=651)^1^ | | | |  |
| --- | --- | --- | --- | --- | --- | --- | --- | --- | --- | --- | --- |
|  |  | **Non-smoker at 180 days** | | | |  | **Non-smoker at 180 days** | | | |  |
|  |  | ***No***  *(n=1258, 96.3%)* | | ***Yes***  *(n=48, 3.7%)* | |  | ***No***  *(n=547, 84.0%)* | | ***Yes***  *(n=104, 16.0%)* | |  |
| **Variable** | **Categories** | **n** | ***%*** | **n** | ***%*** | **p-value** | **n** | ***%*** | **n** | ***%*** | **p-value** |
| **Age On Admission** | 16-24 | 84 | ***6.7*** | <10 | ***-*** | 0.006 | 36 | ***6.6*** | <10 | ***-*** | 0.344 |
|  | 25-39 | 271 | ***21.5*** | <10 | ***-*** |  | 121 | ***22.1*** | 21 | ***20.2*** |  |
|  | 40-59 | 489 | ***38.9*** | 14 | ***29.2*** |  | 218 | ***39.9*** | 36 | ***34.6*** |  |
|  | 60+ | 369 | ***29.3*** | 25 | ***52.1*** |  | 172 | ***31.4*** | 42 | ***40.4*** |  |
|  | *Missing* | *45* | ***3.6*** | *<10* | ***-*** |  | *<10* | ***-*** | *<10* | ***-*** |  |
| **Sex** | Male | 812 | ***64.5*** | 35 | ***72.9*** | 0.333 | 346 | ***63.3*** | 61 | ***58.7*** | 0.437 |
|  | Female | 436 | ***34.7*** | 13 | ***27.1*** |  | 201 | ***36.7*** | 43 | ***41.3*** |  |
|  | *Missing* | *10* | ***0.8*** | *<10* | ***-*** |  | *<10* | ***-*** | *<10* | ***-*** |  |
| **Ethnicity** | Black | 104 | 8.3 | <10 | ***-*** | 0.470 | 97 | 17.7 | 25 | 24 | 0.037 |
|  | Mixed/Asian/Other^2^ | 118 | 9.4 | <10 | ***-*** |  | 88 | 16.1 | <10 | ***-*** |  |
|  | White | 812 | 64.5 | 33 | 68.8 |  | 309 | 56.5 | 65 | 62.5 |  |
|  | Declined or Not stated | 162 | 12.9 | <10 | ***-*** |  | 53 | 9.7 | <10 | ***-*** |  |
|  | *Missing* | *62* | *4.9* | *<10* | ***-*** |  | *<10* | ***-*** | *<10* | ***-*** |  |
| **Intention on admission** | Declined or Withdrawal mgmt.^2^ | 1043 | 82.9 | 24 | 50 | <0.001 | 317 | 58 | 33 | 31.7 | <0.001 |
|  | Quit attempt | 208 | 16.5 | 24 | 50 |  | 223 | 40.8 | 68 | 65.4 |  |
|  | *Missing* | *<10* | ***-*** | *<10* | ***-*** |  | *<10* | ***-*** | *<10* | ***-*** |  |
| **HSI Category** | Low | 134 | ***10.7*** | 12 | ***25*** | 0.009 | 144 | ***26.3*** | 48 | ***46.2*** | 0.004 |
|  | Medium | 447 | ***35.5*** | 12 | ***25*** |  | 243 | ***44.4*** | 40 | ***38.5*** |  |
|  | High | 61 | ***4.8*** | <10 | ***-*** |  | 37 | ***6.8*** | <10 | ***-*** |  |
|  | *Missing* | *616* | ***49*** | *22* | ***45.8*** |  | *123* | ***22.5*** | *12* | ***11.5*** |  |
| **Smoking cessation aids** | None | 512 | ***40.7*** | 11 | ***22.9*** | 0.039 | 251 | ***45.9*** | 64 | ***61.5*** | 0.009 |
|  | Single NRT | 276 | ***21.9*** | 12 | ***25*** |  | 133 | ***24.3*** | 20 | ***19.2*** |  |
|  | Combination | 470 | ***37.4*** | 25 | ***52.1*** |  | 155 | ***28.3*** | 18 | ***17.3*** |  |
|  | *Missing* | *<10* | ***-*** | *<10* | ***-*** |  | *<10* | ***-*** | *<10* | ***-*** |  |
| **Smoking related diagnosis** | Yes | 190 | ***15.1*** | 19 | ***39.6*** | <0.001 | 115 | ***21*** | 30 | ***28.8*** | 0.144 |
|  | No | 728 | ***57.9*** | 18 | ***37.5*** |  | 394 | ***72*** | 70 | ***67.3*** |  |
|  | *Missing* | *340* | ***27*** | *11* | ***22.9*** |  | *38* | ***6.9*** | *<10* | ***-*** |  |
| **Cancer** | Yes | 129 | ***10.3*** | <10 | ***-*** | 0.470 | 30 | ***5.5*** | <10 | ***-*** | 0.388 |
|  | No | 1129 | ***89.7*** | 41 | ***85.4*** |  | 517 | ***94.5*** | 101 | ***97.1*** |  |
| **COPD** | Yes | 330 | ***26.2*** | 24 | ***50*** | 0.001 | 125 | ***22.9*** | 19 | ***18.3*** | 0.366 |
|  | No | 928 | ***73.8*** | 24 | ***50*** |  | 422 | ***77.1*** | 85 | ***81.7*** |  |
| **CVD** | Yes | 191 | ***15.2*** | 16 | ***33.3*** | 0.001 | 55 | ***10.1*** | 13 | ***12.5*** | 0.567 |
|  | No | 1067 | ***84.8*** | 32 | ***66.7*** |  | 492 | ***89.9*** | 91 | ***87.5*** |  |
| **Diabetes** | Yes | 296 | ***23.5*** | 24 | ***50*** | <0.001 | 54 | ***9.9*** | 12 | ***11.5*** | 0.735 |
|  | No | 962 | ***76.5*** | 24 | ***50*** |  | 493 | ***90.1*** | 92 | ***88.5*** |  |
| **Mental Illness** | Yes | 936 | ***74.4*** | 35 | ***72.9*** | 0.950 | 287 | ***52.5*** | 39 | ***37.5*** | 0.007 |
|  | No | 322 | ***25.6*** | 13 | ***27.1*** |  | 260 | ***47.5*** | 65 | ***62.5*** |  |
| **IMD tertile** | Lower | 460 | ***36.6*** | 17 | ***35.4*** | 0.062 | 262 | ***47.9*** | 56 | ***53.8*** | 0.642 |
|  | Middle | 279 | ***22.2*** | 18 | ***37.5*** |  | 159 | ***29.1*** | 25 | ***24*** |  |
|  | Upper | 70 | ***5.6*** | <10 | ***-*** |  | 48 | ***8.8*** | 10 | ***9.6*** |  |
|  | *Missing* | *449* | ***35.7*** | *12* | ***25*** |  | *78* | ***14.3*** | *13* | ***12.5*** |  |

*^1^ Patients who died within 180 days of discharge excluded*

*^2^ Categories collapsed due to sparse data*

### **Additional Table 10:** Source data for regression, stratified by *Hospital*, outcome = *Death by any cause 31 days to 1 year post-discharge* (N=1770)

|  |  | **Hospital A** (N=1125)^1^ | | | |  | **Hospital B** (N=645)^1^ | | | |  |
| --- | --- | --- | --- | --- | --- | --- | --- | --- | --- | --- | --- |
|  |  | **Death 31 days to 1 year post-discharge** | | | |  | **Death 31 days to 1 year post-discharge** | | | |  |
|  |  | ***No***  *(n=1046, 93.0%)* | | ***Yes***  *(n=79, 7.0%)* | |  | ***No***  *(n=615, 95.4%)* | | ***Yes***  *(n=30, 4.7%)* | |  |
| **Variable** | **Categories** | **n** | ***%*** | **n** | ***%*** | **p-value** | **n** | ***%*** | **n** | ***%*** | **p-value** |
| **Age On Admission** | 60+ | 296 | ***28.3*** | 46 | ***58.2*** | <0.001 | 189 | ***30.7*** | 23 | ***76.7*** | <0.001 |
|  | 40-59 | 405 | ***38.7*** | 27 | ***34.2*** |  | 244 | ***39.7*** | <10 | **-** |  |
|  | 16-39 ^2^ | 308 | ***29.4*** | <10 | **-** |  | 182 | ***29.6*** | <10 | **-** |  |
|  | *Missing* | *37* | ***3.5*** | *<10* | ***-*** |  | *<10* | ***-*** | *<10* | ***-*** |  |
| **Sex** | Male | 660 | ***63.1*** | 61 | ***77.2*** | 0.021 | 386 | ***62.8*** | 21 | ***70*** | 0.543 |
|  | Female | 376 | ***35.9*** | 18 | ***22.8*** |  | 229 | ***37.2*** | <10 | **-** |  |
|  | *Missing* | *10* | ***1*** | *<10* | ***-*** |  | *<10* | ***-*** | *<10* | ***-*** |  |
| **Ethnicity** | Black | 86 | ***8.2*** | <10 | **-** | 0.403 | 120 | ***19.5*** | <10 | **-** | 0.313 |
|  | Mixed/Asian/Other^2^ | 97 | ***9.3*** | <10 | **-** |  | 92 | ***15*** | <10 | **-** |  |
|  | White | 659 | ***63*** | 60 | ***75.9*** |  | 345 | ***56.1*** | 22 | ***73.3*** |  |
|  | Declined or Not stated | 147 | ***14.1*** | <10 | **-** |  | 58 | ***9.4*** | <10 | **-** |  |
|  | *Missing* | *57* | ***5.4*** | *<10* | ***-*** |  | *<10* | ***-*** | *<10* | ***-*** |  |
| **Intention on admission** | Declined or Withdrawal mgmt.^2^ | 840 | ***80.3*** | 71 | ***89.9*** | 0.066 | 327 | ***53.2*** | 21 | ***70*** | 0.125 |
|  | Quit attempt | 199 | ***19*** | <10 | **-** |  | 279 | ***45.4*** | <10 | **-** |  |
|  | *Missing* | *<10* | ***-*** | *<10* | ***-*** |  | *<10* | ***-*** | *<10* | ***-*** |  |
| **HSI Category** | Low | 122 | ***11.7*** | <10 | **-** | 0.532 | 183 | ***29.8*** | <10 | **-** | 0.778 |
|  | Medium or High^2^ | 400 | ***38.2*** | 25 | ***31.6*** |  | 305 | ***49.6*** | 13 | ***43.3*** |  |
|  | *Missing* | *524* | ***50.1*** | *49* | ***62*** |  | *127* | ***20.7*** | *11* | ***36.7*** |  |
| **Smoking cessation aids** | None | 416 | ***39.8*** | 28 | ***35.4*** | 0.749 | 294 | ***47.8*** | 20 | ***66.7*** | 0.147 |
|  | Single NRT | 237 | ***22.7*** | 19 | ***24.1*** |  | 144 | ***23.4*** | <10 | **-** |  |
|  | Combination | 393 | ***37.6*** | 32 | ***40.5*** |  | 167 | ***27.2*** | <10 | **-** |  |
|  | *Missing* | *<10* | ***-*** | *<10* | ***-*** |  | *10* | ***1.6*** | *<10* | ***-*** |  |
| **Smoking related diagnosis** | Yes | 157 | ***15*** | 31 | ***39.2*** | <0.001 | 130 | ***21.1*** | 16 | ***53.3*** | <0.001 |
|  | No | 589 | ***56.3*** | 43 | ***54.4*** |  | 443 | ***72*** | 14 | ***46.7*** |  |
|  | *Missing* | *300* | ***28.7*** | *<10* | ***-*** |  | *42* | ***6.8*** | *<10* | ***-*** |  |
| **Cancer** | Yes | 102 | ***9.8*** | 34 | ***43*** | <0.001 | 28 | ***4.6*** | <10 | **-** | 0.001 |
|  | No | 944 | ***90.2*** | 45 | ***57*** |  | 587 | ***95.4*** | 24 | ***80*** |  |
| **COPD** | Yes | 244 | ***23.3*** | 37 | ***46.8*** | <0.001 | 129 | ***21*** | 11 | ***36.7*** | 0.070 |
|  | No | 802 | ***76.7*** | 42 | ***53.2*** |  | 486 | ***79*** | 19 | ***63.3*** |  |
| **CVD** | Yes | 119 | ***11.4*** | 22 | ***27.8*** | <0.001 | 63 | ***10.2*** | <10 | **-** | 0.012 |
|  | No | 927 | ***88.6*** | 57 | ***72.2*** |  | 552 | ***89.8*** | 22 | ***73.3*** |  |
| **Diabetes** | Yes | 215 | ***20.6*** | 35 | ***44.3*** | <0.001 | 59 | ***9.6*** | <10 | **-** | 0.034 |
|  | No | 831 | ***79.4*** | 44 | ***55.7*** |  | 556 | ***90.4*** | 23 | ***76.7*** |  |
| **Mental Illness** | Yes | 741 | ***70.8*** | 63 | ***79.7*** | 0.119 | 300 | ***48.8*** | 18 | ***60*** | 0.311 |
|  | No | 305 | ***29.2*** | 16 | ***20.3*** |  | 315 | ***51.2*** | 12 | ***40*** |  |
| **IMD tertile** | Lower | 369 | ***35.3*** | 47 | ***59.5*** | <0.001 | 293 | ***47.6*** | 18 | ***60*** | 0.183 |
|  | Middle | 226 | ***21.6*** | 18 | ***22.8*** |  | 175 | ***28.5*** | 10 | ***33.3*** |  |
|  | Upper | 59 | ***5.6*** | <10 | **-** |  | 57 | ***9.3*** | <10 | **-** |  |
|  | *Missing* | *392* | ***37.5*** | *11* | ***13.9*** |  | *90* | ***14.6*** | *<10* | ***-*** |  |
| **Smoking status 30-days** | Smoker or unknown | 968 | ***92.5*** | 70 | ***88.6*** | 0.296 | 532 | ***86.5*** | 29 | ***96.7*** | 0.181 |
|  | Non-Smoker | 78 | ***7.5*** | <10 | **-** |  | 83 | ***13.5*** | <10 | **-** |  |

*^1^ Patients who died within 30 days of discharge excluded ^2^ Categories collapsed due to sparse data*

### **Additional Table 11:** Source data for regression, stratified by *Hospital*, outcome = *Readmission 31 days to 1-year post-discharge* (N=1534)

|  |  | **Hospital A** (N=1024)^1^ | | | |  | **Hospital B** (N=510)^1^ | | | |  |
| --- | --- | --- | --- | --- | --- | --- | --- | --- | --- | --- | --- |
|  |  | **Readmission 31 days to 1 year post-discharge** | | | | | **Readmission 31 days to 1 year post-discharge** | | | | |
|  |  | ***No***  *(n=712, 69.5%)* | | ***Yes***  *(n=312, 30.5%)* | |  | ***No***  *(n=412, 80.8%)* | | ***Yes***  *(n=98, 19.2%)* | |  |
| **Variable** | **Categories** | **n** | ***%*** | **n** | ***%*** | **p-value** | **n** | ***%*** | **n** | ***%*** | **p-value** |
| **Age On Admission** | 16-24 | 52 | ***7.3*** | 14 | ***4.5*** | <0.001 | 25 | ***6.1*** | 2 | ***2*** | 0.020 |
|  | 25-39 | 170 | ***23.9*** | 45 | ***14.4*** |  | 97 | ***23.5*** | 12 | ***12.2*** |  |
|  | 40-59 | 260 | ***36.5*** | 135 | ***43.3*** |  | 159 | ***38.6*** | 48 | ***49*** |  |
|  | 60+ | 192 | ***27*** | 115 | ***36.9*** |  | 131 | ***31.8*** | 36 | ***36.7*** |  |
|  | *Missing* | *38* | ***5.3*** | *<10* | ***-*** |  | *<10* | ***-*** | *<10* | ***-*** |  |
| **Sex** | Male | 451 | ***63.3*** | 201 | ***64.4*** | 1.000 | 265 | ***64.3*** | 54 | ***55.1*** | 0.114 |
|  | Female | 251 | ***35.3*** | 111 | ***35.6*** |  | 147 | ***35.7*** | 44 | ***44.9*** |  |
|  | *Missing* | *10* | ***1.4*** | *<10* | ***-*** |  | *<10* | ***-*** | *<10* | ***-*** |  |
| **Ethnicity** | Black | 55 | ***7.7*** | 30 | ***9.6*** | 0.049 | 87 | ***21.1*** | 21 | ***21.4*** | 0.036 |
|  | Mixed/Asian/Other^2^ | 64 | ***9*** | 30 | ***9.6*** |  | 66 | ***16*** | 17 | ***17.3*** |  |
|  | White | 424 | ***59.6*** | 222 | ***71.2*** |  | 218 | ***52.9*** | 59 | ***60.2*** |  |
|  | Declined or Not stated | 105 | ***14.7*** | 30 | ***9.6*** |  | 41 | ***10*** | <10 | **-** |  |
|  | *Missing* | *64* | ***9*** | *<10* | ***-*** |  | *<10* | ***-*** | *<10* | ***-*** |  |
| **Intention on admission** | Declined or Withdrawal mgmt.^2^ | 580 | ***81.5*** | 254 | ***81.4*** | 0.810 | 214 | ***51.9*** | 56 | 57.1 | ***0.437*** |
|  | Quit attempt | 125 | ***17.6*** | 58 | ***18.6*** |  | 192 | ***46.6*** | 41 | 41.8 |  |
|  | *Missing* | *<10* | ***-*** | *<10* | ***-*** |  | *<10* | ***-*** | *<10* | ***-*** |  |
| **HSI Category** | Low | 85 | ***11.9*** | 36 | ***11.5*** | 0.755 | 123 | ***29.9*** | 32 | ***32.7*** | 0.568 |
|  | Medium | 246 | ***34.6*** | 121 | ***38.8*** |  | 176 | ***42.7*** | 43 | ***43.9*** |  |
|  | High | 30 | ***4.2*** | 16 | ***5.1*** |  | 28 | ***6.8*** | <10 | **-** |  |
|  | *Missing* | *351* | ***49.3*** | *139* | ***44.6*** |  | *85* | ***20.6*** | *19* | ***19.4*** |  |
| **Smoking cessation aids** | None | 306 | ***43*** | 108 | ***34.6*** | 0.042 | 202 | ***49*** | 36 | ***36.7*** | 0.072 |
|  | Single NRT | 148 | ***20.8*** | 73 | ***23.4*** |  | 95 | ***23.1*** | 27 | ***27.6*** |  |
|  | Combination | 258 | ***36.2*** | 131 | ***42*** |  | 108 | ***26.2*** | 34 | ***34.7*** |  |
|  | *Missing* | *<10* | ***-*** | *<10* | ***-*** |  | *<10* | ***-*** | *<10* | ***-*** |  |
| **Smoking related diagnosis** | Yes | 96 | ***13.5*** | 66 | ***21.2*** | 0.037 | 84 | ***20.4*** | 22 | ***22.4*** | 1.00 |
|  | No | 388 | ***54.5*** | 179 | ***57.4*** |  | 288 | ***69.9*** | 76 | ***77.6*** |  |
|  | *Missing* | *228* | ***32*** | *67* | ***21.5*** |  | *40* | ***9.7*** | *<10* | ***-*** |  |
| **Cancer** | Yes | 50 | ***7*** | 51 | ***16.3*** | <0.001 | 20 | ***4.9*** | <10 | **-** | 0.953 |
|  | No | 662 | ***93*** | 261 | ***83.7*** |  | 392 | ***95.1*** | 94 | ***95.9*** |  |
| **COPD** | Yes | 136 | ***19.1*** | 128 | ***41*** | <0.001 | 75 | ***18.2*** | 38 | ***38.8*** | <0.001 |
|  | No | 576 | ***80.9*** | 184 | ***59*** |  | 337 | ***81.8*** | 60 | ***61.2*** |  |
| **CVD** | Yes | 65 | ***9.1*** | 65 | ***20.8*** | <0.001 | 36 | ***8.7*** | 20 | ***20.4*** | 0.002 |
|  | No | 647 | ***90.9*** | 247 | ***79.2*** |  | 376 | ***91.3*** | 78 | ***79.6*** |  |
| **Diabetes** | Yes | 128 | ***18*** | 94 | ***30.1*** | <0.001 | 34 | ***8.3*** | 17 | ***17.3*** | 0.012 |
|  | No | 584 | ***82*** | 218 | ***69.9*** |  | 378 | ***91.7*** | 81 | ***82.7*** |  |
| **Mental Illness** | Yes | 467 | ***65.6*** | 269 | ***86.2*** | <0.001 | 189 | ***45.9*** | 66 | ***67.3*** | <0.001 |
|  | No | 245 | ***34.4*** | 43 | ***13.8*** |  | 223 | ***54.1*** | 32 | ***32.7*** |  |
| **IMD tertile** | Lower | 230 | ***32.3*** | 141 | ***45.2*** | <0.001 | 187 | ***45.4*** | 60 | ***61.2*** | <0.001 |
|  | Middle | 150 | ***21.1*** | 73 | ***23.4*** |  | 115 | ***27.9*** | 30 | ***30.6*** |  |
|  | Upper | 35 | ***4.9*** | 13 | ***4.2*** |  | 32 | ***7.8*** | <10 | **-** |  |
|  | *Missing* | *297* | ***41.7*** | *85* | ***27.2*** |  | *78* | ***18.9*** | *<10* | ***-*** |  |
| **Smoking status 30-days** | Smoker or unknown | 659 | ***92.6*** | 293 | ***93.9*** | 0.517 | 363 | ***88.1*** | 88 | ***89.8*** | 0.769 |
|  | Non-Smoker | 53 | ***7.4*** | 19 | ***6.1*** |  | 49 | ***11.9*** | 10 | ***10.2*** |  |

^1^ *Patients who died within year of discharge, readmitted within 30 days, or resident outside of London excluded. ^2^ Categories collapsed due to sparse data*

## **Additional Table 12:** Regression estimates for outcome *Smoking status (non-smoker) at 30 days*

Additional Table 12: Unadjusted and fully adjusted estimates from logistic regression model for outcome: non-smoker at 30 days post-discharge, using imputed analysis, excluding patients who died within 180 days of discharge (n=1957).

| **Outcome** | **Category** | **Unadjusted OR (95% CI)** | **p-value** | **Adjusted OR (95% CI)** | **p-value** |
| --- | --- | --- | --- | --- | --- |
| **Smoking status 30 days** |  |  |  |  |  |
| **Hospital** | **A** | (ref.) |  | (ref.) |  |
|  | **B** | 1.83 (1.35 - 2.49) | <0.001 | 0.91 (0.63 - 1.33) | 0.628 |
| **Age On Admission** | **60+** | (ref.) |  | (ref.) |  |
|  | **40-59** | 0.84 (0.59 - 1.20) | 0.335 | 0.90 (0.59 - 1.37) | 0.627 |
|  | **25-39** | 0.75 (0.48 - 1.15) | 0.184 | 0.77 (0.45 - 1.31) | 0.336 |
|  | **16-24** | 0.90 (0.47 - 1.72) | 0.757 | 0.84 (0.40 - 1.78) | 0.652 |
| **Sex** | **Male** | (ref.) |  | (ref.) |  |
|  | **Female** | 1.11 (0.81 - 1.52) | 0.500 | 1.02 (0.73 - 1.44) | 0.891 |
| **Ethnicity** | **White** | (ref.) |  | (ref.) |  |
|  | **Black** | 1.10 (0.70 - 1.73) | 0.670 | 0.79 (0.47 - 1.32) | 0.364 |
|  | **Mixed/Asian/Other^1^** | 0.65 (0.37 - 1.13) | 0.128 | **0.51 (0.28 - 0.92)** | **0.026** |
| **Intention at first TDS assessment** | **Declined / Withdrawal management^1^** | (ref.) |  | (ref.) |  |
|  | **Quit attempt** | **6.35 (4.60 - 8.77)** | **<0.001** | **6.48 (4.48 - 9.36)** | **<0.001** |
| **Heaviness of Smoking Index** | **Low** | (ref.) |  | (ref.) |  |
|  | **Medium** | **0.51 (0.36 - 0.72)** | **<0.001** | **0.51 (0.34 - 0.77)** | **0.001** |
|  | **High** | **0.44 (0.22 - 0.88)** | **0.021** | 0.47  (0.22 - 1.02) | 0.057 |
| **Smoking cessation aids** | **None** | (ref.) |  | (ref.) |  |
|  | **Single** | 1.17 (0.79 - 1.71) | 0.435 | 1.00 (0.65 - 1.53) | 1.000 |
|  | **Combination** | 1.02 (0.72 - 1.46) | 0.893 | 0.94 (0.61 - 1.46) | 0.791 |
| **Primary diagnosis** | **Smoking related** | **1.79 (1.26 - 2.53)** | **0.001** | **1.49 (1.00 - 2.23)** | **0.052** |
| **Past diagnoses** | **Cancer** | 1.08 (0.62 - 1.78) | 0.772 | 0.85 (0.47 - 1.53) | 0.583 |
|  | **COPD** | 0.81 (0.56 - 1.16) | 0.267 | **0.61 (0.39 - 0.96)** | **0.032** |
|  | **CVD** | 1.21 (0.79 - 1.80) | 0.371 | 1.31 (0.80 - 2.13) | 0.283 |
|  | **Diabetes** | 0.97 (0.65 - 1.41) | 0.881 | 1.22 (0.78 - 1.90) | 0.388 |
|  | **Mental & behav.** | **0.53 (0.39 - 0.72)** | **<0.001** | 0.79 (0.55 - 1.12) | 0.185 |
| **IMD tertile** | **Lower** | (ref.) |  | (ref.) |  |
|  | **Middle** | 1.43 (0.97 - 2.12) | 0.072 | **1.55 (1.01 - 2.38)** | **0.045** |
|  | **Upper** | **1.77 (1.00 - 3.13)** | **0.049** | 1.70 (0.90 - 3.23) | 0.104 |

*^1^Categories collapsed due to sparse data*

## **Additional Table 13**: Regression estimates for outcome *Smoking status (non-smoker) at 90 days*

Additional Table 13: Unadjusted and fully adjusted estimates from logistic regression model for outcome: non-smoker at 90 days post-discharge, using imputed analysis, excluding patients who died within 180 days of discharge (n=1957).

| **Outcome** | **Category** | **Unadjusted OR (95% CI)** | **p-value** | **Adjusted OR (95% CI)** | **p-value** |
| --- | --- | --- | --- | --- | --- |
| **Smoking status 90 days** |  |  |  |  |  |
| **Hospital** | **A** | (ref.) |  | (ref.) |  |
|  | **B** | 2.12 (1.53 - 2.92) | <0.001 | 1.17 (0.80 - 1.72) | 0.420 |
| **Age On Admission** | **60+** | (ref.) |  | (ref.) |  |
|  | **40-59** | 0.71 (0.49 - 1.03) | 0.073 | 0.91 (0.59 - 1.41) | 0.684 |
|  | **25-39** | 0.62 (0.39 - 0.98) | 0.039 | 0.82 (0.47 - 1.43) | 0.478 |
|  | **16-24** | 0.57 (0.27 - 1.22) | 0.147 | 0.62 (0.27 - 1.46) | 0.278 |
| **Sex** | **Male** | (ref.) |  | (ref.) |  |
|  | **Female** | 0.92 (0.65 - 1.28) | 0.605 | 0.80 (0.55 - 1.15) | 0.219 |
| **Ethnicity** | **White** | (ref.) |  | (ref.) |  |
|  | **Black** | 1.19 (0.75 - 1.90) | 0.459 | 0.91 (0.54 - 1.55) | 0.736 |
|  | **Mixed/Asian/Other^1^** | 0.56 (0.30 - 1.04) | 0.068 | **0.44 (0.23 - 0.87)** | **0.018** |
| **Intention at first TDS assessment** | **Declined / Withdrawal management^1^** | (ref.) |  | (ref.) |  |
|  | **Quit attempt** | **4.85 (3.49 - 6.74)** | **<0.001** | **4.52 (3.10 - 6.59)** | **<0.001** |
| **Heaviness of Smoking Index** | **Low** | (ref.) |  | (ref.) |  |
|  | **Medium** | **0.49 (0.35 - 0.70)** | **<0.001** | **0.52 (0.35 - 0.78)** | **0.002** |
|  | **High** | **0.40 (0.19 - 0.85)** | **0.017** | 0.46 (0.21 - 1.03) | 0.060 |
| **Smoking cessation aids** | **None** | (ref.) |  | (ref.) |  |
|  | **Single** | 1.09 (0.73 - 1.61) | 0.685 | 0.98 (0.64 - 1.52) | 0.942 |
|  | **Combination** | 0.82 (0.56 - 1.19) | 0.288 | 0.82 (0.52 - 1.29) | 0.388 |
| **Primary diagnosis** | **Smoking related** | **2.25 (1.58 - 3.19)** | **<0.001** | **1.78 (1.19 - 2.67)** | **0.005** |
| **Past diagnoses** | **Cancer** | 1.23 (0.70 - 2.04) | 0.438 | 0.90 (0.50 - 1.63) | 0.737 |
|  | **COPD** | 1.20 (0.84 - 1.70) | 0.300 | 1.01 (0.64 - 1.57) | 0.981 |
|  | **CVD** | 1.27 (0.81 - 1.92) | 0.274 | 1.07 (0.65 - 1.77) | 0.782 |
|  | **Diabetes** | 1.22 (0.82 - 1.77) | 0.309 | 1.50 (0.96 - 2.34) | 0.078 |
|  | **Mental & behav.** | **0.47 (0.34 - 0.65)** | **<0.001** | **0.67 (0.47 - 0.97)** | **0.034** |
| **IMD tertile** | **Lower** | (ref.) |  | (ref.) |  |
|  | **Middle** | **1.59 (1.06 - 2.39)** | **0.025** | **1.83 (1.18 - 2.84)** | **0.007** |
|  | **Upper** | **1.94 (1.09 - 3.46)** | **0.024** | **1.99 (1.06 - 3.75)** | **0.033** |

*^1^Categories collapsed due to sparse data*

## Sensitivity analyses: comparison of regression estimates from primary and sensitivity models

### **Additional Figure 2:** Plot comparing estimates from primary and sensitivity regression models for outcome *Accepted intervention at first TDS assessment*


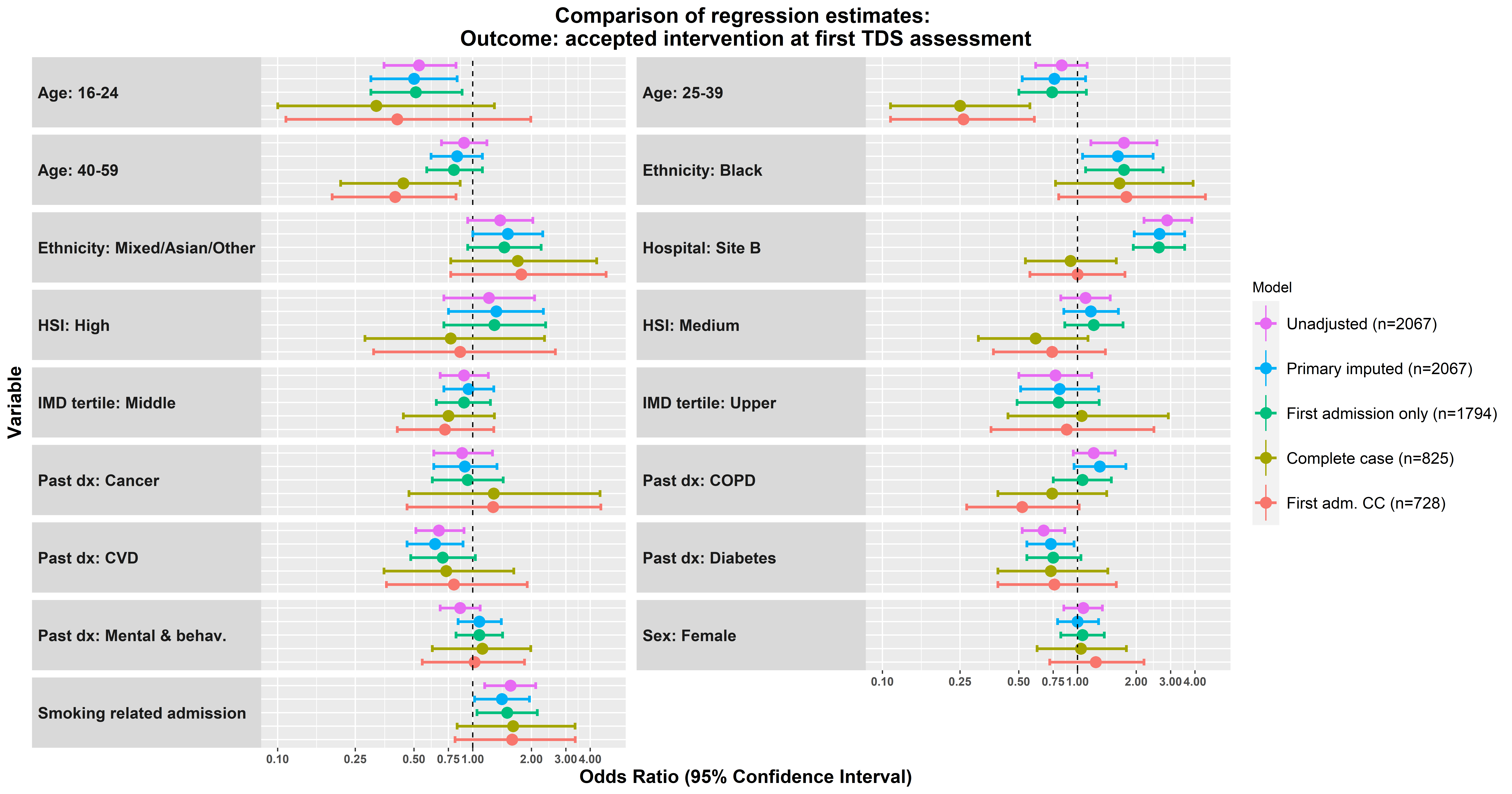


### **Additional Figure 3:** Plot comparing estimates from primary and sensitivity regression models for outcome *Smoking status (non-smoker) at 30 days*


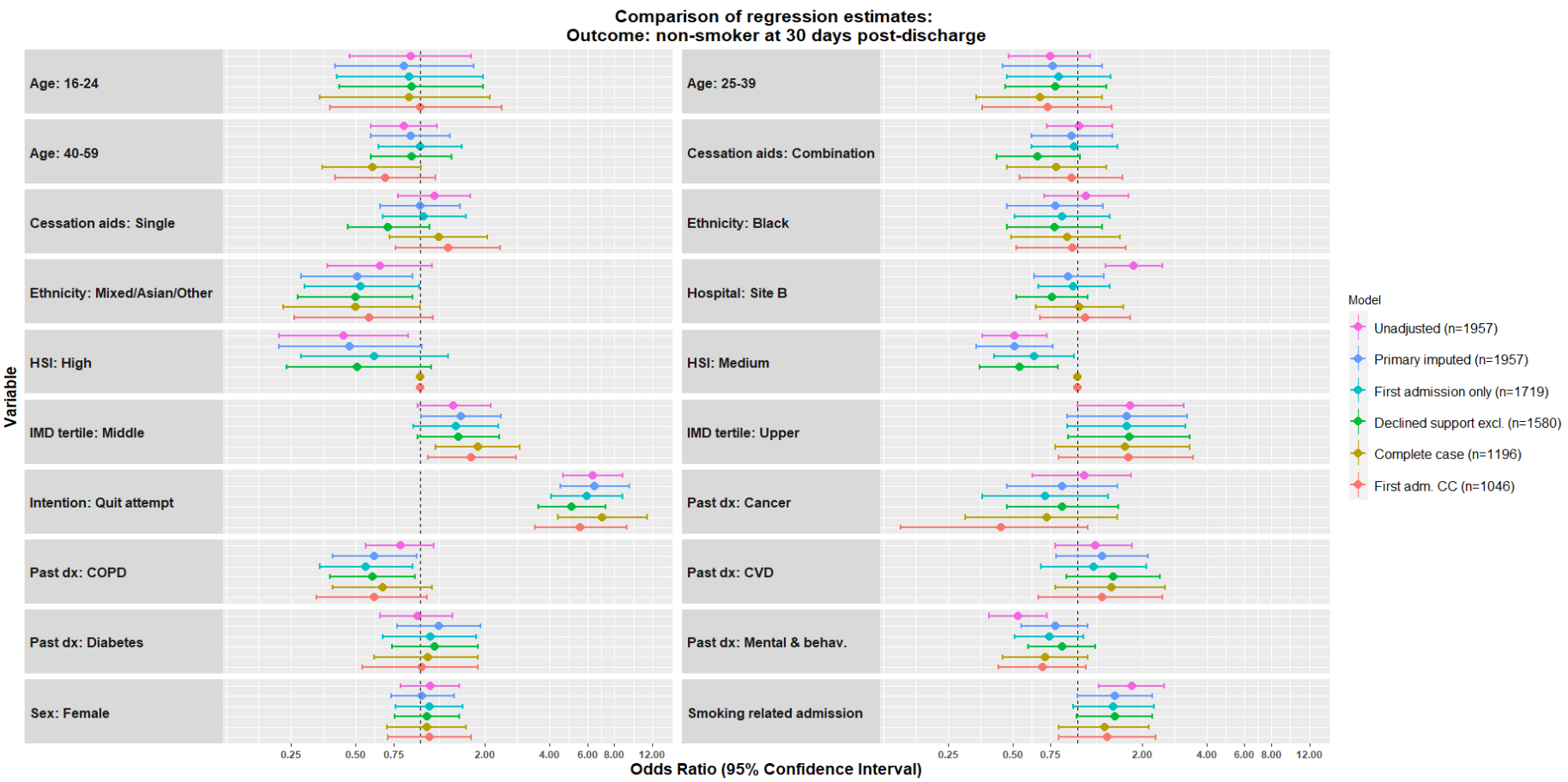


Declined support excl. = Sample restricted to patients who accepted support at first TDS assessment.

First adm. CC = Complete case analysis of first admission only data

*HSI not included in complete case analyses due to excessive amount of missing data*

### **Additional Figure 4:** Plot comparing estimates from primary and sensitivity regression models for outcome *Smoking status (non-smoker) at 90 days*


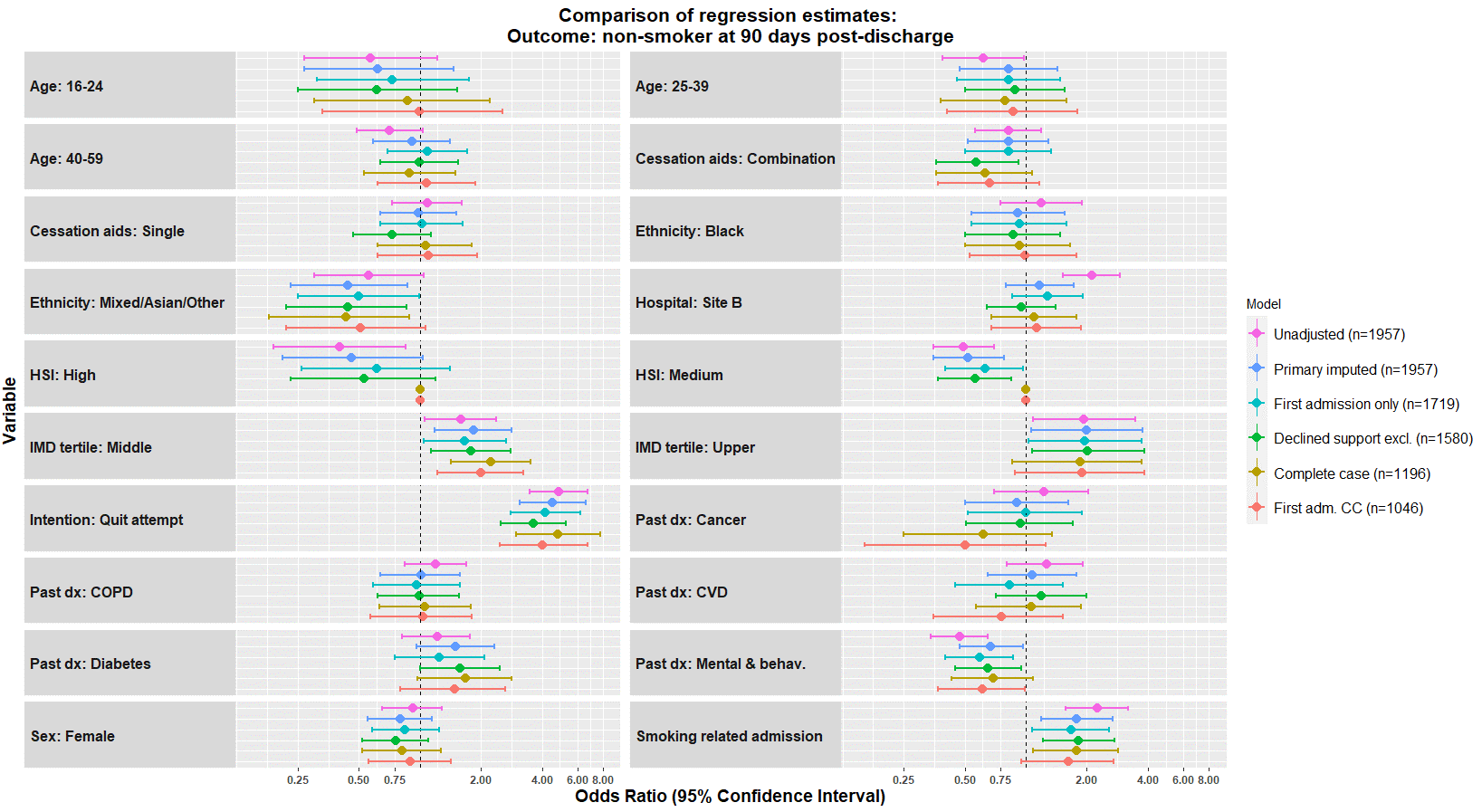


Declined support excl. = Sample restricted to patients who accepted support at first TDS assessment.

First adm. CC = Complete case analysis of first admission only data

*HSI not included in complete case analyses due to excessive amount of missing data*

### **Additional Figure 5:** Plot comparing estimates from primary and sensitivity regression models for outcome *Smoking status (non-smoker) at 180 days*


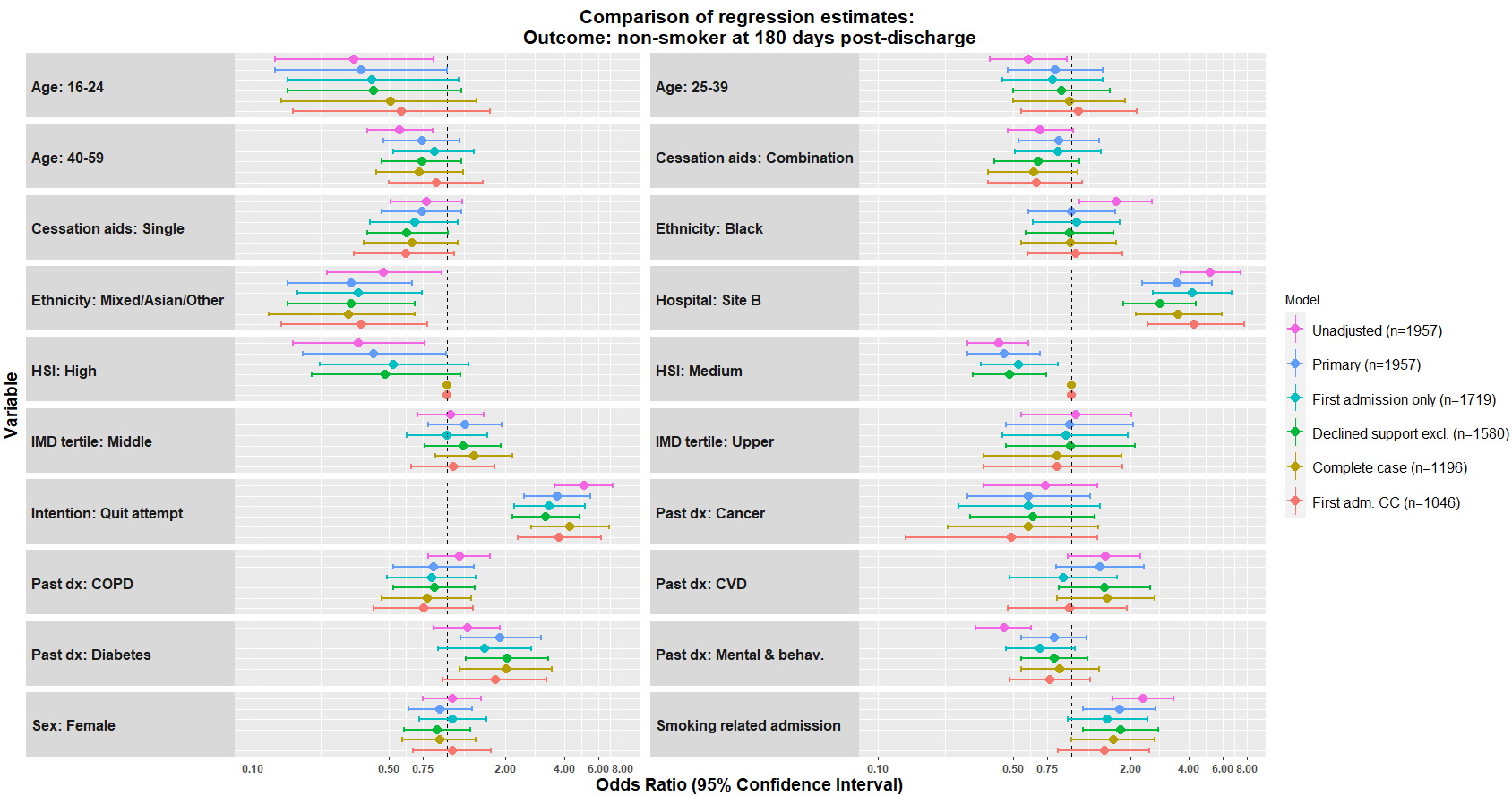


Declined support excl. = Sample restricted to patients who accepted support at first TDS assessment.

First adm. CC = Complete case analysis of first admission only data

*HSI not included in complete case analyses due to excessive amount of missing data*

### **Additional Figure 6:** Plot comparing estimates from primary and sensitivity regression models for outcome *All-cause death between 31 days and 1-year*


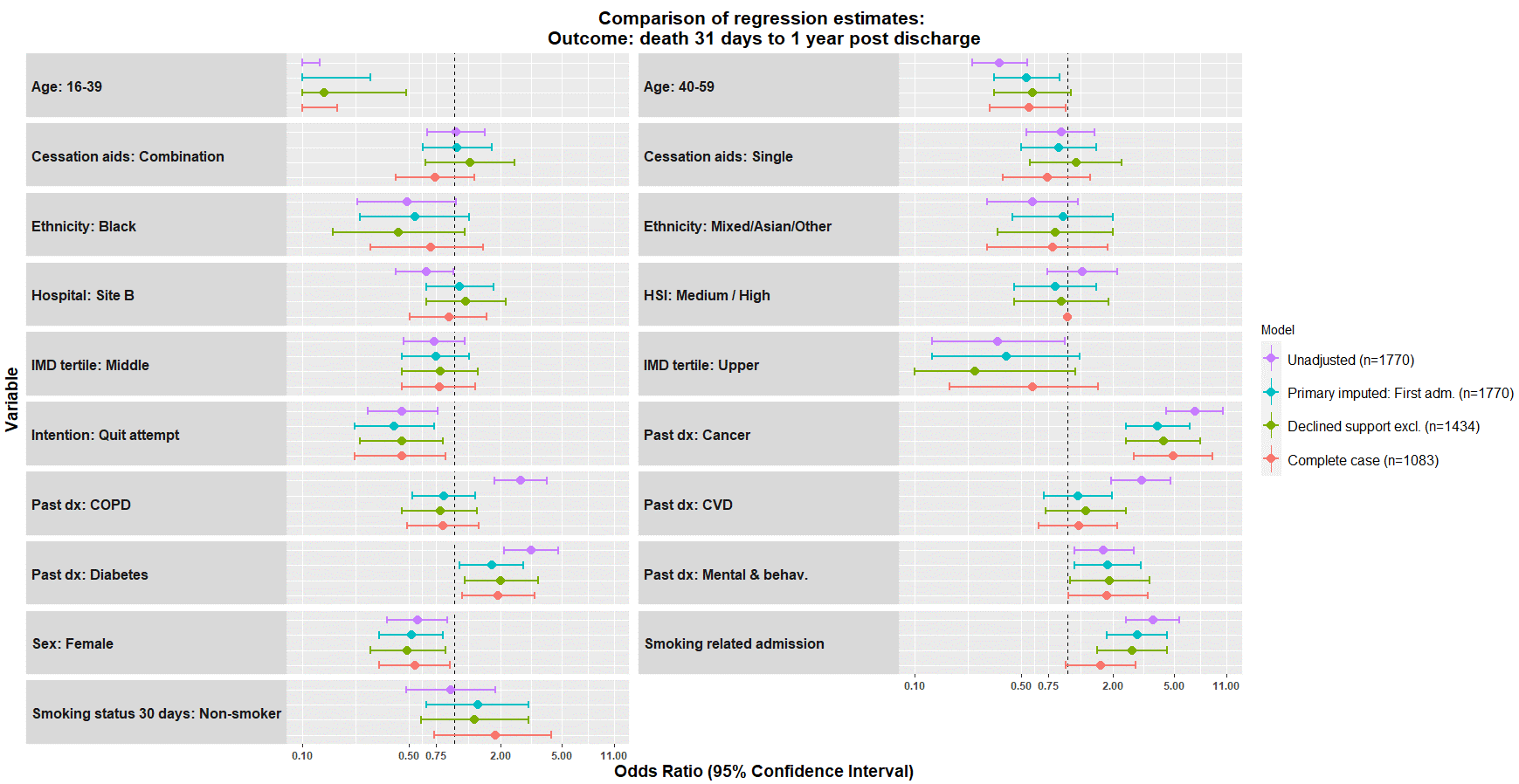


Declined support excl. = Sample restricted to patients who accepted support at first TDS assessment.

*Primary analysis restricted to first admission only to prevent double counting of deaths*

*HSI not included in complete case analyses due to excessive amount of missing data*

### **Additional Figure 7:** Plot comparing estimates from primary and sensitivity regression models for outcome *All-cause readmission between 31 days and 1-year*


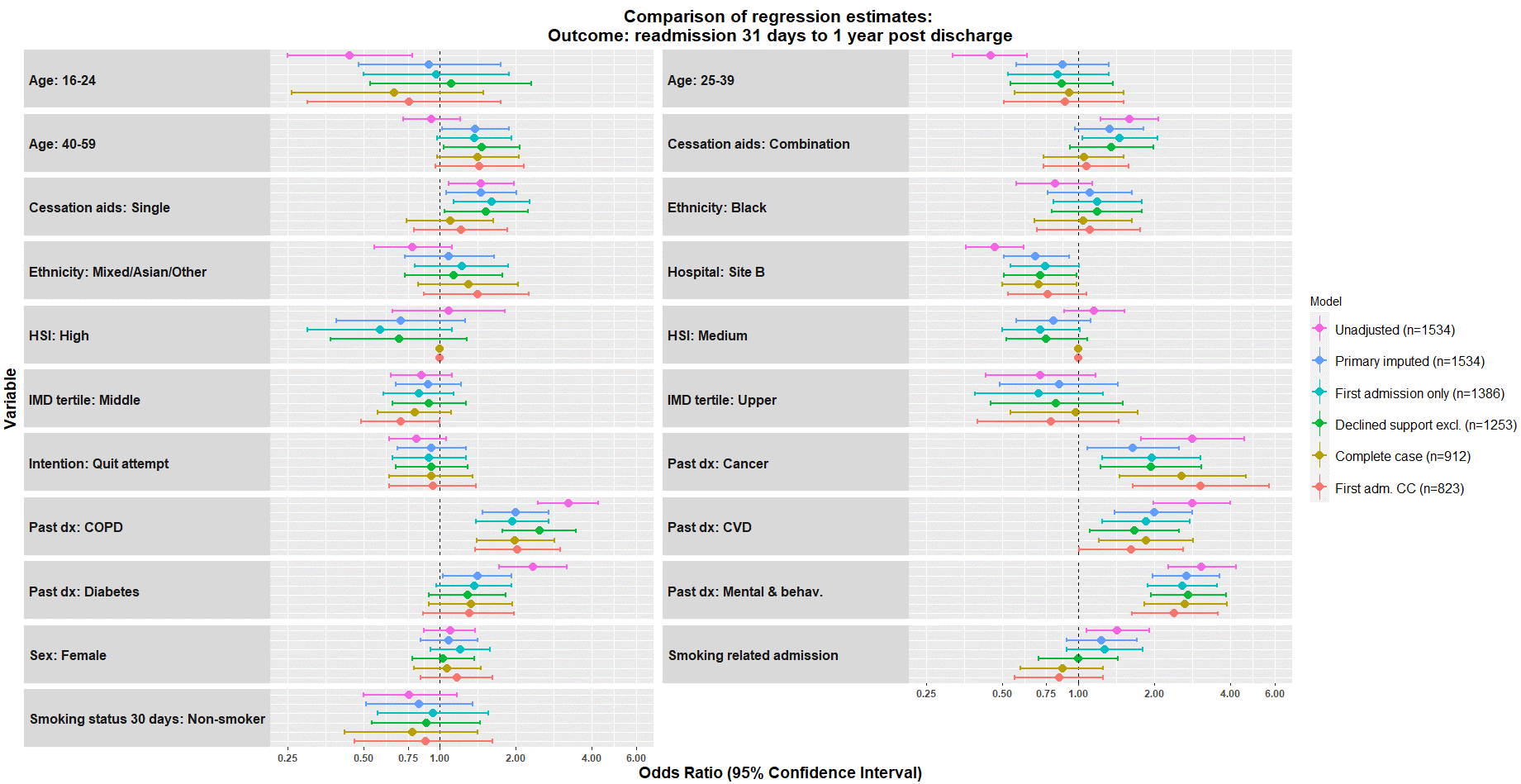
Patients who lived outside of London excluded, as well as patients who died within year of discharge, and patients who were readmitted within 30 days

*HSI not included in complete case analyses due to excessive amount of missing data*

*Declined support excl. = Sample restricted to patients who accepted support at first TDS assessment.*

First adm. CC = Complete case analysis of first admission only data
